# Assessing the Ecophysiology of Methanogens in the Context of Recent Astrobiological and Planetological Studies

**DOI:** 10.3390/life5041652

**Published:** 2015-12-03

**Authors:** Ruth-Sophie Taubner, Christa Schleper, Maria G. Firneis, Simon K.-M. R. Rittmann

**Affiliations:** 1 Research Platform: ExoLife, University of Vienna, Türkenschanzstraße 17, 1180 Vienna, Austria; 2 Institute of Astrophysics, University of Vienna, Türkenschanzstraße 17, 1180 Vienna, Austria; 3 Archaea Biology and Ecogenomics Division, Department of Ecogenomics and Systems Biology, University of Vienna, Althanstraße 14, 1090 Vienna, Austria

**Keywords:** Archaea, extremophiles, metabolism, Solar System, methanogenesis, space, microorganism, Mars, Earth, icy moons

## Abstract

Among all known microbes capable of thriving under extreme and, therefore, potentially extraterrestrial environmental conditions, methanogens from the domain Archaea are intriguing organisms. This is due to their broad metabolic versatility, enormous diversity, and ability to grow under extreme environmental conditions. Several studies revealed that growth conditions of methanogens are compatible with environmental conditions on extraterrestrial bodies throughout the Solar System. Hence, life in the Solar System might not be limited to the classical habitable zone. In this contribution we assess the main ecophysiological characteristics of methanogens and compare these to the environmental conditions of putative habitats in the Solar System, in particular Mars and icy moons. Eventually, we give an outlook on the feasibility and the necessity of future astrobiological studies concerning methanogens.

## 1. Introduction

Methane (CH${}_{4}$), the smallest hydrocarbon molecule, is frequently detected in different anoxic environments as well as in the atmosphere of Earth, and its presence has also been verified on other Solar System bodies, e.g., on Saturn’s icy moons Titan [1] and Enceladus [2] or on Mars [3,4,5]. On Earth, CH${}_{4}$ is currently contributing to global warming, because it is a potent greenhouse gas, but nowadays it is also one of the most important fossil fuels for human society. Most of the CH${}_{4}$ present on Earth is of biogenic origin [6] and there is still the elusive possibility that also CH${}_{4}$ detected on other Solar System bodies could be of biological origin. To date, the only organisms known to be capable of producing CH${}_{4}$ as a side product of their energy conserving metabolism are methanogenic microorganisms (methanogens) [7].

Methanogens are an intriguing group of prokaryotes from the domain Archaea. Initially, Archaea were often considered being extremophilic microorganisms with adaptations to high temperature or high salt. However, the importance of archaea contributing to biogeochemical cycles in extremophilic, but also in non-extremophilic habitats, on Earth is tremendous [8]. Within the domain archaea methanogens so far only belong exclusively to the phylum Euryarchaeota, one of the currently five established archaeal phyla [8], but metagenomic sequencing revealed a putative methanogenic micoroganism in the phylum Bathyarchaeota [9].

Methanogens are divided into five classes (Methanobacteria, Methanococci, Methanomicrobia, Methanopyri, and Thermoplasmata) and seven orders (Methanobaceriales, Methanococcales, Methanomicrobiales, Methanosarcinales, Methanocellales, Methanopyrales, and Methanomassiliicoccales) [6,8,10]. The seventh order of methanogens (Methanomassiliicoccales) has only recently been discovered and belongs to the class Thermoplasmata [11,12]. All yet characterized methanogens are known to be obligate anaerobic chemolithoautotrophs or chemolithoheterotrophs capable of producing CH${}_{4}$ in order to acquire energy in a process referred to as methanogenesis [6,10,11,13,14,15,16], from the following substrates:

(1) from hydrogen (H${}_{2}$) and carbon dioxide (CO${}_{2}$) or from carbon monoxide (CO) or from formate (HC$O_{2}^{-}$)
(2) from methanol, methylamines, methanethiol or methylsulfide
(3) from acetate
(4) or from methanol (CH${}_{3}$OH), methylamines and methylsulfide as methyl group donor and H${}_{2}$ as electron source.

From a phylogenetic perspective, methanogens putatively resemble one of the oldest life forms having emerged on Earth. Furthermore, membrane lipids of putative methanogenic origin were detected in fossils dating 2.7 billions years back in Earth’s history [17]. Moreover, also CH${}_{4}$ of putative biological origin, and therefore of possibly methanogenic origin, was detected in samples dating 3.7 billion years back in time [18]. Regarding the ability of methanogens for fixing CO${}_{2}$ into cellular carbon biological methanogenensis could have emerged as first metabolic reactions next to hydrothermal vent systems [19]. However, this view is still under investigation and discussion [20,21].

Methanogens can be found in almost every anoxic environment, like in swamps, deep soil sediments, peat bogs, sediments, permafrost soils, anaerobic digester, sediments of aquatic environments, and in the intestine of half of the human population as well as in animals [6,10,22,23,24,25,26,27,28,29,30,31,32,33,34,35,36,37]. Methanogens play an important role in Earth’s global carbon cycle where they are involved in the last step of biomass breakdown [6,10]. Moreover, methanogens are of relevance to agriculture in general and to livestock production in particular, because CH${}_{4}$ emissions from industrialized animal production farms and from wetland crop production (e.g., rice fields) are substantially contributing to an increase in global warming. Furthermore, methanogens are interesting study objects because they can withstand extreme environmental conditions [38,39].

Methanogens are utilized biotechnologically for biogas production in anaerobic digesters or for anaerobic wastewater treatment. In the former process methanogens perform the final step in anaerobic digestion of organic waste or biomass by producing CH${}_{4}$ [6,37]. Currently methanogens are being explored to be applied in biological CH${}_{4}$ production processes, referred to as biological methanation or biomethanation. Therein hydrogenotrophic methanogens are examined to be used for high rate volumetric CH${}_{4}$ production from CO${}_{2}$ and H${}_{2}$ and/or microbiological (bio)gas upgrading [13,40,41,42,43,44].

**Figure 1:**
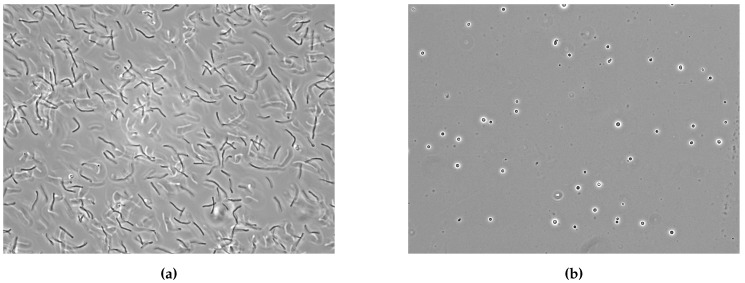
Phase contrast images of *Methanothermobacter marburgensis* and *Methanosarcina soligelidi*. (**a**) Phase contrast image (magnification 1000×) of *M. marburgensis* DSM 2133. Specimen was obtained in late exponential phase from a fed-batch fermentation. The cells are rod shaped and elongated; (**b**) Phase contrast image (magnification 1000×) of *M. soligelidi* DSM 26065. Specimen was obtained from a closed batch cultivation. The cells are coccoid.

Astonishing morphological features of methanogens include, e.g., the methanochondroitin sheath of certain methanosarcia species [45], but typical morphologies of methanogens are shown in Figure 1.

Hitherto more than 150 pure cultures of methanogens are described [46,47] exhibiting extraordinary biochemical, morphological, physiological, metabolic and biotechnological features [6,10,11,13,14,40,42,43,44,48,49,50,51]. Due to their ubiquitous presence, their metabolic versatility and their ability to endure multiple extreme environmental conditions methanogens are being considered in astrobiological research.

In the last decade, new findings about extrasolar planetary systems, icy moons in the Solar System, but also the newly explored capabilities of extremophiles led to a rethinking of the extent of the classical habitable zone [52,53]. Various attempts have been made to define new potential niches of life in the Solar System, e.g., in the concept of the “life supporting zone” [54]. This is a generalization of Kasting’s concept of a habitable zone, where alternative solvents—*i.e.*, other than water or solvents of water/ammonia—determine their own habitable zones around a star. Nevertheless, the detection of liquid water in the outer Solar System opens up entirely new areas far beyond the water snow line [55] where life-as-we-know-it could be situated.

Of astrobiological interest are those Solar System bodies on which we assume at least local areas that could fulfil the physical and chemical requirements that methanogens could propagate in an extraterrestrial environment. This includes the residues of near-surface melt water on Mars and subsurface oceans or local seas on various icy moons like Europa or Enceladus.

Mars, the fourth planet in the Solar System, seems to have possessed a huge amount of liquid water on its surface in the Noachian period [56] and therefore could have been a habitable planet in its “early” years. Due to its low atmospheric pressure, there cannot be liquid water on its surface currently. Nevertheless, there are indications for subsurface aquifers [57]. Methanogens would perfectly fit into this subsurface environment especially due to their ecophysiological capabilities to tolerate and endure extreme conditions. Several studies focus on the potential of methanogens to grow under Martian-like environmental conditions [58,59,60,61,62,63,64,65,66,67,68,69,70,71]. The detection of subsurface water aquifers on the icy moons in the outer Solar System extended the group of potential habitats by far. However, at the moment, methanogens and sulfur-reducing bacteria seem to be the only terrestrial microbes that could propagate in such an environment (see Section 3).

In this contribution we outline the main ecophysiological characteristics of methanogens. We compare these characteristics of methanogens to the environmental conditions of their putative habitats in the Solar System, in particular to Mars and icy moons. We will point out their potential capability to be able to inhabit extraterrestrial biospheres in the planetary system. We present an overview concerning studies of methanogens with an astrobiological relevance and we present our conclusions about the role of methanogens for the search for extraterrestrial life in the Solar System. Eventually, we give an outlook on the feasibility and the necessity of future astrobiological studies with these microbes.

## 2. Ecophysiological Characteristics of Methanogens

Methanogens are well known for a variety of astonishing morphological and ecophysiological features. One of these ecophysiological features is their ability to grow hydrogenotrophically while varying their growth-to-product-yield Y${}_{\left(x/CH4\right)}$—a mechanism also referred to as uncoupling [6,10,40,44,72,73,74,75,76,77,78]. Uncoupling in methanogens occurs when environmental factors (*i.e.*, pH, temperature, reducing compounds, H${}_{2}$ concentrations) become sub- or superoptimal or if they change. As a consequence of changing environmental factors methanogens react rapidly by altering their metabolism redirecting the carbon flux to either biomass formation or to maintaining cellular homoeostasis. As an example it has been found that *Methanobacterium thermoautotrophicum* responds to variations in H${}_{2}$ concentration by differentially expressing methenyl-tetrahydromethanopterin dehydrogenase and methyl coenzyme M reductase isoenzymes [77]. Other examples include the varying Y${}_{\left(x/CH4\right)}$ in *Methanothermobacter marburgensis* upon exposure to different ammonia concentration or dilution rates [40,44]. Also phosphate limitation [72] and iron- or H${}_{2}$-limited cultures [73] as well as temperature [78] were shown to uncouple Y${}_{\left(x/CH4\right)}$. In their natural environment methanogens are occasionally confined to a shallow reaction zone where they might achieve only little net growth in order to be able to propagate or gain energy for metabolic reactions [79].

We will review studies on methanogens in relation to their ecophysiological adaptations to different temperature zones. Furthermore, we will highlight the main ecophysiological and metabolic features of methanogens that allow them to grow under different amounts of radiation, osmolarities, pH values, and desiccation.

### 2.1. Temperature

**Figure 2:**
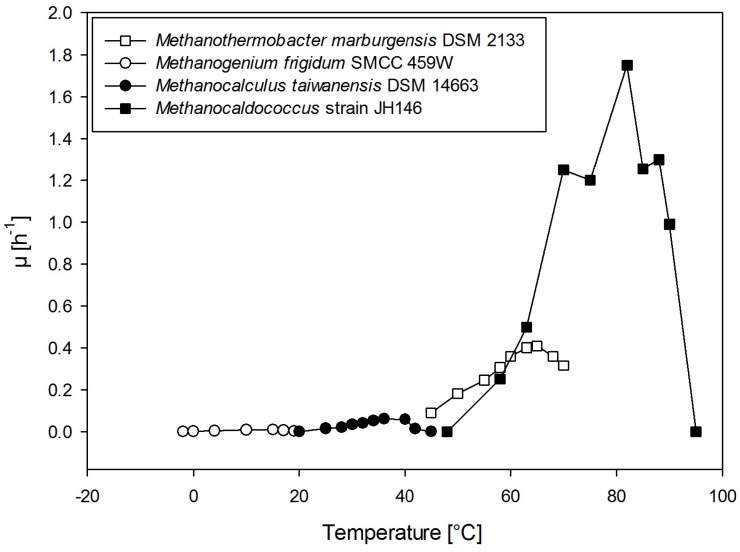
A plot of specific growth rates (*μ*) of four methanogenic strains. The four strains were arbitrarily selected independent of the specific metabolism as well as independent of growth conditions, but to span the entire temperature range from psychrophilic to hyperthermophilic growth conditions. Each single data point has been carefully retrieved from literature; for *M. marburgensis* DSM 2133 [82], for *M. frigidum* SMCC 459W [33], for *M. taiwanensis* DSM 14663 [83], for *Methanocaldococcus* strain JH146 [78]. Interestingly, with increasing growth temperature maximum *μ* values were found to increase.

Methanogens span a huge temperature range with organisms that are psychrophilic, mesophilic, thermophilic, and even hyperthermophilic [22,23,24,25,26,27,28,29,30,31,32,33,34,35,80] (see Figure 2). Recently a novel hydrogenotrophic methanogen, “*Candidatus* Methanoflorens stordalenmirensis” was identified to be frequently present in permafrost wetland and members of *Candidatus* familiy “Methanoflorentaceae” are widespread in permafrost and appear to be key mediators of CH${}_{4}$ emissions in cold but seasonally thawing environments [36]. Another psychrophilic strain, *Methanosarcina* sp. SMA-21 (now referred to as *M. solegelidi* SMA-21) was shown to tolerate freezing and survival of 98.5% in comparison to, e.g., *Methanobacterium* sp. MC-20 exhibiting only 1% survival [81]. Furthermore, the temperature-dependent starvation tolerance compared among different *Methanosarcina* species and *M. solegelidi* SMA-21 was shown to comprise a high survival potential at 4 ${}^{\circ }$C and at 28 ${}^{\circ }$C compared to the two other methanogens tested [81].

Although individual strains of methanogens comprise a temperature window for growth of approximately 45 ${}^{\circ }$C, the main biochemical methanogenesis pathways are not restricted to a certain temperature. However, pathways of methanogenesis are functional under conditions spanning a temperature range from below 0 ${}^{\circ }$C [38] up to the hottest ever determined metabolic reactions of any organism described, as high as 122 ${}^{\circ }$C [80]. The methanogen exhibiting the highest ever measured growth temperature is *Methanopyrus kandleri* strain 116. This organism is able to grow at 122 ${}^{\circ }$C [80].

Outer Solar System bodies, which could putatively support methanogenic life, are situated in the temperature range of psychrophiles [84] (especially in the subsurface water reservoirs) or even colder (see Section 3, Table 3). Therefore, especially psychrophilic methanogens should be of great interest to astrobiological research. However, the temperature can be locally higher, especially near potential hydrothermal vents.

In order to distinguish different levels of psychrophily, psychrophilic methanogens are classified according to their temperature niche, which is narrow or wide [38,85]. For this reason the terms “stenopsychrophile” and “eurypsychrophile” have been introduced [38,86,87]. Stenopsychrophiles are considered true psychrophiles, which can only thrive in a narrow temperature range and cannot be cultivated when exposed to elevated temperatures. In contrast, eurypsychrophiles can tolerate higher temperatures and comprise a higher mean optimum growth temperature compared to stenopsychrophiles. Psychrophilic methanogens have already been the topic of many research endeavours, because they play an important role in 75% of Earth’s habitats [38,85]. A summary of psychrophilic methanogenic strains can be found in Table 1.

Mechanisms for temperature adaptation in methanogens were identified on different cellular and structural levels. At the protein level cold adaptation mechanisms were examined in *Methanococcoides burtonii*. Elongation factor 2 (EF2) has been found to be active at low but to be unstable at high growth temperatures [88,89,90]. Furthermore, EF2 interacting proteins of *M. burtonii* but also compatible solutes are involved in activating as well as stabilizing the protein machinery under low growth temperatures [90]. In a study by Lim *et al*. a putative DEAD-box RNA helicase gene (deaD) was identified in *Methanococcoides burtonii* that was abundantly expressed at 4 ${}^{\circ }$C [91].

**Table 1:** Summary of currently known psychrophilic strains and their main temperature and pH features.

Strain	Temp. [${}^{\circ }$C]			pH			Ref.
	min.	opt	max	min.	opt.	max.	
*Methanospirillum psychrodurum*	4	25	32	6.5	7	8	[92]
*Methanosarcina baltica*	3	21	28	6.3	7.2	7.5	[32]
*Methanosarcina lacustris*	1	25	35	4.5	7	8.5	[93]
*Methanolobus psychrophilus*	0	18	25	6	7–7.2	8	[94]
*Methanogenium marinum*	5	25	25	5.5	6–6.6	7.7	[95]
*Methanogenium frigidum*	0	15	17	6.3	7.5–7.9	8	[33]
*Methanohalobium evestigatum*	50	n.a. ${}^{1}$	n.a.	n.a.	7.4	n.a.	[96]
*Methanogenium cariaci*	15	20–25	35	6	6.8–7.2	7.5	[97]
*Methanogenium boonei*	5	19.4	25.6	6.4	n.a.	7.8	[98]
*Methanoculleus marisnigri*	15	20–25	48	6	6.2–6.6	7.6	[99]
*Methanoculleus chikugoensis*	15	25	40	6.7	6.7–7.2	8	[100]
*Methanococcoides alaskense*	2.3	23.6	28.4	6.3	n.a.	7.5	[101]
*Methanococcoides burtonii*	1.7	23.4	29.5	6.8	n.a.	8.2	[102]
*Methanospirillum stamsii*	5	20–30	37	6.0	7.0–7.5	10	[31]
*Methanosarcina soligelidi*	0	28	54	4.8	7.8	9.9	[34]
*Candidatus* “Methanoflorens stordalenmirensis”	n.a.	n.a.	n.a.	n.a.	n.a.	n.a.	[36]

${}^{1}$ not available.

A similar observation was made in the hyperthermophilic archaeon *Thermococcus kodakarensis* where a homologous DEAD-box helicase gene TK0306 (Tk-DeaD) was induced under suboptimal growth temperatures [103] suggesting that this DEAD-box helicase is generally expressed as a result of cold stress. Different from cold shifts of thermophiles the methanogen *M. burtonii* did not show decreased modifications of its tRNAs. Like in bacteria the organism generally exhibited few modifications, in particular dihydrouridine incorporation into tRNA.

Furthermore, a genome comparison of two cold-adapted archaea, *Methanogenium frigidum* and *M. burtonii*, was done in order to identify genomic characteristics distinguishing these two-species from other organisms. Predicted and modelled proteins from *M. frigidum* and *M. burtonii* comprise a higher quantity of non-charged polar amino acids in the solvent-accessible protein area. Hence, glutamin and threonin were detected in higher abundance in the proteins. Also a lower content of hydrophobic amino acids, particularly leucin, could be revealed [104]. Furthermore, Saunders *et al*. identified a cold shock domain (CSD) protein (CspA homolog) in *M. frigidum* as well as two hypothetical proteins with CSD-folds and a unique winged helix DNA-binding domain protein in *M. burtonii* [104]. In another study, a proteomics approach was employed to analyze the functional characteristics of *Methanosarcina barkeri* in a low-temperature shock response (from 37 ${}^{\circ }$C to 15 ${}^{\circ }$C) or for its low-temperature adaptation strategies at 15 ${}^{\circ }$C. By applying a combined approach of proteomics and growth studies insights into the low-temperature adaptation capacity of *M. barkeri* could be revealed [105]. Above mentioned findings indicate that mechanisms on the genome level (e.g., expression of deaD at suboptimal growth temperature) as well as on the proteome level (e.g., activity of EF2) distinguish stenopsychrophilic, eurypsychrophilic as well as (hyper-)thermophilic methanogens regarding their ecophysiologcal adaptations forced to grow at suboptimal temperatures.

Another adaptation mechanism of methanogens to growth temperature variation is their ability to modify membrane lipids in order to maintain membrane fluidity, *i.e.*, the lipid membrane of organisms has to be kept in the liquid crystalline phase in order to stay functional. Generally, the archaeal lipid membrane was found to stay in the liquid crystalline phase between 0 to 100 ${}^{\circ }$C [106]. In order to maintain membrane fluidity psychrophilic methanogens have adapted growth temperature mediated lipid unsaturation by selective saturation through the action of geranylgeranyl reductase instead of applying unsaturation mechanisms occurring in bacteria [107]. On the other hand, a recent survey indicated that the ether lipid membrane composition in general, or its unsaturation properties of methanogens in particular, are not a clear indicator whether the organism is adapted to a life in cold or hot environments [106]. For example the lipid membrane of *M. thermoautotrophicus*, growing at 65 ${}^{\circ }$C, contains both archaeol and the membrane spanning caldarchaeol as do some methanogens growing at 37 ${}^{\circ }$C. Moreover, the core membrane lipids of *M. kandleri*, growing at 90 ${}^{\circ }$C, is archaeol [106]. However upon increasing growth temperature of *Methanocaldococcus jannaschii* from 45 ${}^{\circ }$C to 65 ${}^{\circ }$C the lipid membrane composition changes from initially, mainly archaeol containing lipid membranes, to cyclic archaeol as well as caldarchaeol [106,108]. Interestingly, the presence of double bonds in isoprenoid chains also does not indicate an adaptation to lower growth temperatures [106].

### 2.2. Pressure

Methanogens are also fascinating organisms due to their ability to tolerate and to grow at low-pressure as well as under overpressure conditions. In some of their natural environments (e.g., sea floor) methanogens have to tolerate >20 MPa overpressure [28,29,79]. Cultivation of methanogens under moderate overpressure conditions (up to 300 kPa) can be performed in closed batch fermentation in serum bottles [109] as well as in bioreactors [43,110]. Cultivation of methanogens under low pressure conditions and at pressures beyond 300 kPa requires special equipment for cultivation [58,59,111,112,113]. Low-pressure experiments are relevant for astrobiological research, because on some Solar System bodies (e.g., Mars, see Section 4.1), compared to Earth, lower above ground pressure occurs, which is due to special planetary and atmospheric characteristics.

Two different methanogenic strains were examined under moderate overpressure conditions, up to 300 kPa, regarding their growth and CH${}_{4}$ production kinetics using bioreactors in continuous culture and under fed-batch conditions [42,43,110]. Cultivation of the thermophilic methanogen KN-15 was examined under pressurized fed-batch as well as under continuous culture conditions. In fed-batch culture, both, the point at which biomass growth kinetics changed from exponential growth to linear growth as well as *μ* increased with an increase of overpressure [110]. Results for the cultivation of *M. marburgensis* showed that CH${}_{4}$ production is substrate limited in relation to gas availability [43]. Although applying overpressure the maximum physiological capacity could not be reached. However, unfortunately, no experiments were conducted yet aiming to elucidate the intracellular response to substrate limitation in *M. marburgensis*.

*M. jannaschii* was cultivated under overpressure as well as under gas limited conditions. Growth only occurred if gas availability was sufficient and it was found that *M. jannaschii* exhibited a stress response under both, overpressure and low-pressure cultivation conditions. Moreover, pressure induced a response on the transcriptional level in addition to a substrate limitation stress [113].

Overpressure and decompression experiments were performed using *M. jannaschii* in a specially equipped high-pressure bioreactor [111]. Upon rapid decompression from approximately 26 MPa to atmospheric pressure, *M. jannaschii* cell envelopes ruptured. However, when the decompression time was increased from 1 s to 5 min the rupture of *M. jannaschii* cell envelopes significantly decreased [111]. Additional overpressure and high-temperature investigations using *Methanococcus thermolithotrophicus* were accomplished in 10 mL nickel tubes and applying a set of autoclaves connected in series. This set-up enabled fast (10 min) temperature and overpressure changes of 400 ${}^{\circ }$C and 400 MPa, respectively [114]. While overpressure of 50 MPa was found to be optimal for the growth of *M. thermolithotrophicus* applying overpressure of >75 MPa resulted in increased cell lysis as well as in changes of morphology and propagation characteristics [114]. Furthermore, *M. jannaschii* was studied under overpressure conditions by applying helium or argon and H${}_{2}$/CO${}_{2}$ at different temperatures. At both 86 ${}^{\circ }$C and 90 ${}^{\circ }$C growth and CH${}_{4}$ production of *M. jannaschii* became accelerated at up to 75 MPa overpressure, and Y${}_{\left(x/CH4\right)}$ was uncoupled above 90 ${}^{\circ }$C. The high-temperature limit for CH${}_{4}$ production of *M. jannaschii* was found to be increased by increasing pressure, but not if H${}_{2}$/CO${}_{2}$ (4:1 ratio) was applied [112].

The response to an increase in CO${}_{2}$ pressure on methanogens has been inconsistently examined in microcosms experiments mimicking a biocoenosis found in deep subsurface oil reservoir formations (55 ${}^{\circ }$C, 5 MPa). Methanogenesis was found to occur under both, overpressure as well as low-pressure CO${}_{2}$ conditions. By increasing CO${}_{2}$ pressure the rate of methanogenesis was accelerated and, depending on the pressure of CO${}_{2}$, different methanogenic pathways were found to be activated [115].

These adaptations towards low- and also high-pressure reveal that methanogens can survive and may be cultivated under a variety of growth conditions including multi-factorial stress conditions (e.g., substrate limitation, pressure) [10,44,58] and they respond to environmental changes in plentiful metabolic adaptations such as the utilization of different methanogenic pathways as well as the activation of overflow metabolism due to variations of CO${}_{2}$ and CO partial pressure [15,16,115]. Due to their potential to adapt to changing and sometimes extreme environmental conditions, methanogens seem to be ideal candidates for astrobiological studies (see Section 4).

### 2.3. pH

Organisms adapted to extremely acidic conditions are found among bacteria, archaea and fungi. Many hyperthermophilic archaea of the family *Sulfolobaceae* may thrive at pH values between 1 and 4.5 [116,117,118] and strains from the euryarchaeotal order Thermoplasmatales are capable of growing even below pH = 0 [8]. However, the record holder regarding growth at acidic pH is *Picrophilus oshimae* [119].

To date most of the characterized methanogens, which were obtained in pure culture, preferentially grow at neutral pH values. Astonishingly, some methanogens, *i.e.*, *Methanocaldococcus* sp. and *M. marburgensis* exhibit a broader pH range niche ranging into the acidic pH conditions [44,78]. Moreover, there are other methanogens known to be able to grow at acidic pH [98,120]. However, only recently, a natronophilic methanogen has been isolated exhibiting growth at pH values between 8.0 to 10.2 with a pH optimum between 9.0–9.5 [121]. A summary of methanogens cultivable in either acidic or alkaline conditions is presented in Table 2.

**Table 2:** Summary of currently known methanogens cultivable in either acidic or alkaline conditions.

Strain	Temp. [${}^{\circ }$C]			pH			Ref.
	min.	opt.	max	min.	opt.	max.	
*Methanospirillum stamsii*	5	20–30	37	6	7–7.5	10	[31]
*Methanocalculus natronophilus*	14	30–37	45	8	9–9.5	10.2	[121]
*Methanospirillum hungatei*	20	37–45	50	6.5	7–9	10	[12]
*Methanobrevibacter millerae*	33	36–42	43	5.5	7–8	10	[122]
*Methanobrevibacter olleyae*	28	28–42	42	6	7.5	10	[122]
*Methanotorris igneus*	45	88	91	5	5.7	7.5	[123]
*Methanosphaerula palustris*	14	30	35	4.8	5.5	6.4	[120]
*Methanoregula boonei*	10	35–37	40	4.5	5.1	5.5	[98]

### 2.4. Osmolarity

Methanogens accumulate compatible solutes in order to reduce the difference between osmotic potentials between the cytoplasm and the environment. Compatible solutes are small osmoprotective molecules which do not alter cellular processes, even if they are intracellularly concentrated up to high molarities. All methanogens are dependent on low salt concentrations in order to maintain cellular integrity and homoeostasis [26,124].

The main compatible solute of methanogens was found to be trimethylglycine (glycine betaine) [125], herein referred to as betaine. Betaine can be accumulated intracellularly in high quantities in, e.g., *Methanosarcina thermophila* TM-1 [126]. *M. thermophila* TM-1 was furthermore shown to be adaptable to different osmolarities either by synthesis of *α*-glutamate and N-*ε*-acetyl-*β*-lysine, by accumulation of betaine or by accumulating potassium ions. Betaine can be accumulated through an uptake system consisting of a single, high-affinity H${}^{+}$ and/or Na${}^{+}$ driven transporter [126].

By applying different NaCl concentrations (from 0.05 to 1.0 mol·L${}^{-1}$) the effect of osmolarity on growth kinetics as well as on changes of morphology of different *Methanosarcina* species was investigated [127]. Between 0.4 to 1.0 mol·L${}^{-1}$ NaCl the outer surface methanochondroitin layer, which is a characteristic feature of the multicellular aggregated stage of *Methanosarcina* spp., disaggregated and *Methanosarcina* spp. were found growing as single cells. All *Methanosarcina* spp. strains tested, comprised of a methanochondroitin layer, exhibited enhanced stability at <0.2 mol·L${}^{-1}$ NaCl osmolarity and grew at higher temperatures [127]. In another experimental set-up different NaCl concentrations of 0.7 to 3.4 mol·L${}^{-1}$ were applied to a methanogen growth medium resulting in accumulation of K${}^{+}$. Furthermore, *β*-glutamate was detected when methanogens were grown in a medium containing NaCl at concentrations <1.5 mol·L${}^{-1}$ [128].

Another interesting characteristic of halotolerant methanogens was identified in *Methanogenium marinum* AK-1, a strain isolated from permanently cold marine sediments at Skan Bay (Alaska, USA). *M. marinum* AK-1 was found to grow in salinities of 0.25–1.25 mol·L${}^{-1}$ Na${}^{+}$. The authors concluded that *M. marinum* AK-1 and methanogens from similar saline habitats are capable of metabolizing at low pressures of H${}_{2}$ (<1 Pa), but, however, this was found to be dependent on the partial pressure of CH${}_{4}$ [95].

Recently, a novel methanogenic strain, *Methanocalculus natronophilus* Z-7105T was isolated from the bottom sediments of a collector next to a soda lake (Tanatar II, Altai, Russia) [121]. The pH spectrum of the strain was already discussed before, but interestingly *M. natronophilus* was found to be obligatory dependent on carbonates. An optimum carbonate concentration between 0.7–0.9 mol·L${}^{-1}$ enabled growth of *M. natronophilus* as well as Na${}^{+}$ (but not Cl${}^{-}$ ions) were obligatory required at optimal concentrations of 1.4–1.9 mol·L${}^{-1}$, too. The organism thus can be regarded as osmophil and halotolerant and it should be interesting to study osmo-adaptation mechanisms in *M. natronophilus* [121] and other methanogens.

Adaptation to high salt concentrations was achieved with the freshwater methanogen *Methanosarcina mazei* Gö1. It was able to tolerate 1 mol·L${}^{-1}$ salt by uptake of betaine from the growth medium through the osmoprotectant transporter A (OpuA), whose expression was confirmed to be salt inducible [129].

In *Methanohalophilus portucalensis* FDF1 also *α*-glutamate and the beta-amino acids *β*-glutamine as well as N-*ε*-acetyl-*β*-lysine have been described to act as osmoprotective substances in methanogens [128,130]. Again betaine is preferentially taken up from the growth medium instead of being synthesised *de novo* like other osmoprotective compounds such as *β*-glutamine and N-*ε*-acetyl-*β*-lysine [130]. Recently, however, an adenosine derivate has been proposed as a novel compatible solute of *Methanolobus psychrophilus* R15 [85]. Further studies suggest that some of the aforementioned compatible solutes might also be considered as cryoprotective compounds [85].

The role of other compatible solutes, including single species ions, towards higher growth temperature adaptation in methanogens would still require elucidation.

### 2.5. Ionizing and UV Radiation

Life on Earth is protected from cosmic and Solar radiation through the motion of Earth’s liquid core inducing a protective shield referred to as magnetosphere. Furthermore, most of the UV irradiation is blocked from reaching Earth’s surface through the ozone layer molecules. However, the situation is completely different on, e.g., the Martian surface, because Mars is constantly exposed to UV radiation due to the lack of a significant ozone layer [131] and cosmic radiation will only be blocked in low quantity because of a weak and irregular magnetosphere [132].

Regarding the effect of UV damage on the ecophysiology and molecular biology of archaea only a limited number of studies have been published [133,134,135,136,137]. However, only two short and in some parts inconclusive conference reports are available focussing on the survival of methanogens upon exposure to UV light [138,139]. The knowledge generated from UV light experiments with methanogens is still too limited to describe general ecophysiological trends for methanogens confronted with ionizing and/or UV-radiation.

### 2.6. Desiccation

On the surface and possibly also in the subsurface of Mars as well as in different environments on Earth, e.g., deserts and salt formations, extremely dry conditions prevail [140]. Halophilic archaea were detected in salt rock by using culture independent and culture dependent methods [141]. Results from the cultivation of pure strains show cell survival rates between 0.1%–10% following freezing and embedding in salt crystals, indicating that low water activity and low temperature can be survived by halophilic archaea [140,142,143].

Not only halophilic archaea but also methanogens were investigated regarding their tolerance towards desiccation in order to determine their survival capacity. *Methanosarcina mazei* TM1, *Methanobacterium formicicum* and *Methanobrevibacter aboriphilicus* could recover from low water activity (a${}_{w}$) when exposed to air or when kept under nitrogen atmosphere [144]. Moreover, soil had a protective effect on some methanogens when exposed to desiccation and air [144]. In another approach four methanogenic strains, *M. wolfeii*, *M. barkeri*, *M. formicicum* and *M. maripaludis* were applied in desiccation experiments in order to examine survival periods when incubated under different pressure conditions [58]. *M. barkeri*, *M. wolfeii* and *M. formicicum* survived 330, 180 and 120 days of desiccation at 10 kPa, respectively. *M. maripaludis* did not survive desiccation at 10 kPa. However, *M. wolfeii*, *M. barkeri* and *M. formicicum* survived desiccation for 120 days at 60 Pa, but *M. maripaludis* survived 60 days at 60 Pa [58].

*M. soligelidi* SMA-21 (formerly known as *Candidatus Ms. gelisolum*) was isolated from permafrost and exhibited astonishing survival capacity towards freeze-drying and desiccation as well as when being exposed to air. The strain grows optimally at 28 ${}^{\circ }$C, pH = 7.8 and 0.02 mol·L${}^{-1}$ NaCl. To examine desiccation cell suspensions were put onto microscope cover slides, which were exposed to aerobic conditions or completely dried. *M. soligelidi* survived up to 25 days of desiccation as well as exposure to air for 72 h [34,81]. Recently, three methanogenic strains, *M. soligelidi*, *M. mazei* and *Methanobacterium movilense*, were shown to produce CH${}_{4}$ when incubated in a regolith-like matrix lacking nutrients. Moreover, the survival of the three methanogens was analyzed after a 400 day desiccation period [145]. All the three tested methanogens could be reactivated, and CH${}_{4}$ production could be observed, after the designated incubation period ended. However, especially *M. mazei* and *M. movilense* showed higher CH${}_{4}$ production after reactivation from of 400 days of desiccation in different Mars regolith-like matrix analogues, as compared to *M. soligelidi*.

The aforementioned results indicate that especially methanogens of the genus *Methanosarcina*—*M. mazei*, *M. barkeri*, *M. soligelidi*—exhibit extraordinary physiological features making them promising organisms for astrobiological research endeavours.

## 3. Known and Potential Habitats for Methanogens in the Solar System

**Figure 3:**
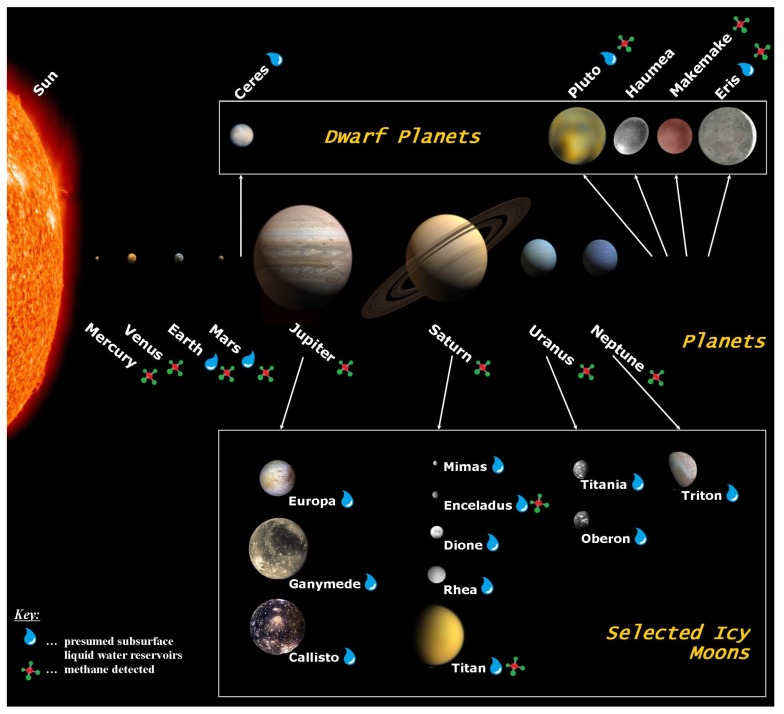
Artist’s conception of the Solar System highlighting confirmed or modelled presence of CH${}_{4}$ and liquid water on different Solar System bodies. The main focus of our study lies on Mars and the icy moons Enceladus, Europa, and Titan. The sizes within each of the three presented groups of Solar System bodies (planets, dwarf planets, and icy moons) are to scale. The distances between the bodies are not to scale; adapted from [146,147,148,149].

In this section we outline known (Earth) and potential habitats for methanogens in the Solar System. For the selection of these habitats we focused on those which may host liquid water and on which CH${}_{4}$ has been detected. An overview of the selected Solar System bodies is given in Table 3 and Figure 3.

**Table 3:** Parameters of Mars, the Moon, and selected moons and icy bodies presented in this review- the last column shows if the potential subsurface aquifer is in direct contact with the underlying silicate core/mantle.

	Host Planet	R [km]	*ρ* [kg m${}^{-3}$]	T${}_{\mathrm{surface}}$ [K]			Atmosphere	Water/Core
				$T_{\min}$	$T_{\mathrm{mean}}$	$T_{\max}$		
Moon	Earth	1737.4 ± 1 [150]	3344 ± 5 [151]	26 ${}^{1}$ [152]	220 [153]	390 ${}^{2}$	no	no
Mars	-	3389.50 ± 0.2 [150]	5.5134 ± 0.0006 [154]	150 ${}^{3}$ [155]	215 [155]	290 ${}^{4}$ [155]	yes [155]	-
Ceres	-	476.2± 1.7 [150]	2077 ± 36 [156]	n.a.	n.a.	n.a.	no	no (?)
Ganymede	Jupiter	2631.2 ± 1.7 [150]	1942 ± 5 [151]	70 [157]	109 [153]	152 [157]	no	no/yes ${}^{5}$
Callisto	Jupiter	2410.3 ± 1.5 [150]	1834 ± 4 [151]	80±5 [158]	134 [153]	165 [158]	yes (thin)	no ${}^{6}$
Europa	Jupiter	1560.8 ± 0.3 [150]	3013 ± 5 [151]	50 [153]	103 [153]	n.a.	yes (thin)	yes
Titan	Saturn	2574.73 ± 0.09 [150]	1882 ± 1 [151]	n.a.	94 [159]	n.a.	yes	no (?)
Enceladus	Saturn	252.1 ± 0.2 [160]	1609 ± 5 [160]	32.9 [161]	75 [161]	≥157 [162]	local [162]	yes (?) [163]
Dione	Saturn	561.4 ± 0.4 [150]	1476 ± 4 [151]	n.a.	n.a.	n.a.	no	n.a.
Mimas	Saturn	198.2 ± 0.4 [150]	1150 ± 4 [151]	n.a.	n.a.	n.a.	no	no
Triton	Neptune	1352.6 ± 2.4 [150]	2059 ± 5 [151]	n.a.	39 [155]	n.a.	yes (thin)	yes (?)
Pluto	-	1195 ± 5 [150]	2030±60 [164]	35 [165]	44	55 [165]	yes (thin)	yes (?)

${}^{1}$ at Hermite crater, coldest temperature measured anywhere in the Solar System [152]; ${}^{2}$ at the equator; ${}^{3}$ at the poles in winter; ${}^{4}$ at latitudes $\pm 60^{\circ }$ in summer at midday; ${}^{5}$ Present studies indicate that Ganymede may host several alternate ice and liquid water layers with the lowest layer in contact to the rocky mantle [166] (see Figure 6b); ${}^{6}$ According to NASA’s Galileo gravimetric data, Callisto is only partly differentiated which results in a mantle/core-structure being a mixture of ice and rocky material.

### 3.1. Earth

By now, Earth is the only known habitat for methanogens and generally life-as-we-know-it. Methanogens may have already appeared in the Archaean era (before 2.5 Gyr ago) [17,18], where the CH${}_{4}$ in the fluid inclusions found in approximately 3.5-Gyr-old hydrothermal precipitates from Pilbara craton, Australia, is discussed to be of abiotic origin [167]. Methanogens are known to be widely distributed in nearly all habitats on Earth as already discussed in the previous section. Most of the CH${}_{4}$ produced on Earth is of biotic origin, but there exist also abiotic processes that produce CH${}_{4}$ like serpentinization or Fischer-Tropsch synthesis [168]. To distinguish between CH${}_{4}$ of biogenic and thermogenic (*i.e.*, abiogenic) origin, the isotopic ratios have to be evaluated. Therefore, if we could measure the isotopic ratios of CH${}_{4}$ on another celestial body, this would be a hint towards the presence of methanogens on this body. Furthermore, abiogenic CH${}_{4}$ shows a near absence of C${}_{2}$ and higher alkane [169].

### 3.2. Mars

Mars is the outermost terrestrial planet in our Solar System and lies just outside the classical habitable zone around the Sun. Its radius is about half and its mass about one-tenth of the Earth’s. It has a thin atmosphere consisting of 96% CO${}_{2}$, 1.93% argon (${}^{40}$Ar) and 1.89% molecular nitrogen (N${}_{2}$) along with traces of molecular oxygen (O${}_{2}$) and CO [170]. The mean atmospheric pressure on the surface is about 0.6 kPa [155] and the scale height of the atmosphere is about 10.8 to 11.1 km [171,172] (compared to the Earth’s scale height of 8.5 km [173]). The temperature varies between 310 K in summer (at noon at the equator) and 140 K in winter (at the poles), with an average temperature of 215–218 K [155].

Until the beginning of the last century, Mars was thought to be probably inhabited [174]. When Giovanni Schiaparelli observed Mars in 1882, he discovered a vast amount of linear features on the surface which were named *canali*. These *canali* had been interpreted as (artificial) waterways or even irrigation canals built by a supposed intelligent civilization on Mars. It took until the 1960s, when the first Mariner spacecraft visited Mars and refuted the idea of intelligent life on this planet. Up to now, dozens of Mars missions with orbiters, landers, and even rovers have not been able to detect definite signs for extant or extinct life.

Nevertheless, according to current knowledge, there was a wet period in Mars’ history. According to a study of Villanueva *et al.*, in the Noachian period (about 4 billion years ago) almost half of Mars’ northern hemisphere was covered by an ocean with depths of more than 1.6 km [175]. Due to this huge amount of liquid water, it is rather unlikely that the whole mass of water of the ancient oceans have been vaporized and been lost to space, but that there are subsurface water reservoirs or transient residues of near-surface melt water. However, today, we can find water just in the form of ice or, according to the latest findings of NASA’s Mars Reconnaissance Orbiter, in brine flows [176]. So far, the majority of water ice has been found at the polar ice caps, but there are also buried ice deposits at nonpolar latitudes [177].

During the last decades there have been several reports about the detection of CH${}_{4}$ on Mars. In 1969, George Pimentel announced in a press conference the detection of CH${}_{4}$ for the first time. According to his presentation, NASA’s Mariner 7 mission had detected CH${}_{4}$ near Mars’ south polar cap [178]. Just one month later the findings had to be retracted due to a misinterpretation of the data [179]. In the following years, announcements on detections of CH${}_{4}$ on the one hand (e.g., ESA’s Mars Express Orbiter in 2004 [3], NASA’s Infrared Telescope Facility and the W.M. Keck telescope in 2009 [4], or NASA’s Curiosity Rover in 2015 [5]) and proofs to the contrary (e.g., NASA’s Curiosity Rover in 2013 [180]) on the other hand alternated periodically (for a critical review, see [181]). However, if there is CH${}_{4}$ in the Martian atmosphere, its amount is very low (mean value of 0.69 ± 0.25 parts per billion by volume (ppbv) [5]). For comparison, the concentration of CH${}_{4}$ in Earth’s atmosphere amounts roughly to 1800 ppbv [181]. Another interesting aspect of the various reports on CH${}_{4}$ detections is that there seems to be a seasonal variation in the CH${}_{4}$ concentration [182]. The authors report that in the warmest season higher concentrations of CH${}_{4}$ were found. This could imply that the amount of volatile release or/and the amount of potential biological activity is higher due to the higher available energy. However, it may indicate as well a systematic analysis error from ground based observations that are seasonally dependent.

**Figure 4:**
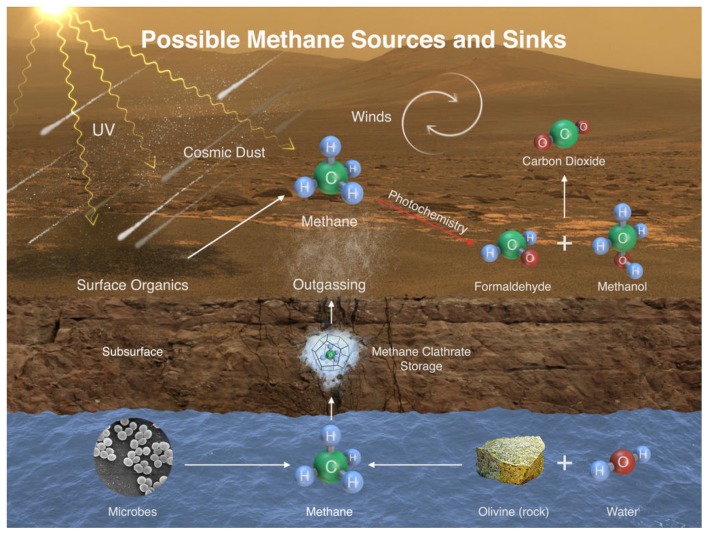
Possible sinks and sources of CH${}_{4}$ on Mars (PIA19088), Image credit: NASA/JPL- Caltech/ SAM-GSFC/Univ. of Michigan.

In general, CH${}_{4}$ on Mars can have three principal origins [183]: volcanism, biological activity like methanogenesis, or the condensation from the solar nebula. The latter would require a transport mechanism from Mars’ interior to the outside, *i.e.*, a geological process which would be associated with volcanism (*i.e.*, the first mentioned potential source). Furthermore, CH${}_{4}$ in the Martian atmosphere is extremely unstable. There are several potential sinks like its decay to CO${}_{2}$ due to photochemical effects or undefined surface oxidation phenomena (see Figure 4). With lifetimes ranging from less than a year to up to 350 years for photochemical processes in the Martian atmosphere [182], CH${}_{4}$ must be permanently produced. To sum up, if we detect a time-varying amount of CH${}_{4}$ on Mars without any evidence for an ongoing geological activity, this could be an indirect sign for life on this planet.

Life on Mars would have to resist strong UV radiation due to the lack of an ozone layer [184]. However, life on early Earth was likely resistant to UV and therefore potential Martian microbes may possess resistance to this type of radiation as well [133]. Furthermore, the low surface temperature, low pressure, and desiccation on Mars’ surface may act as an inhibitor for life forms, such that they could rather thrive in the subsurface water reservoirs which would be sheltered from radiation. Several studies have been performed to test the growth and survival of methanogens under Mars-like conditions (see Section 4.1).

### 3.3. Potential Habitable Moons and Small Icy Bodies

The findings of the last decades, that icy moons may harbour a large amount of liquid water under their ice shells have opened new horizons in the rising field of Astrobiology. Nevertheless, liquid water alone may be sufficient, but not necessary for icy moons to be habitable. Another necessary condition may be that the liquid water reservoir is in contact with the silicate core or seafloor to get heated by hydrothermal activity, radioactive decay, *etc.*, and to receive other compounds essential for life by various processes like, e.g., serpentinization. The processes between the ocean and the underlying rocky layers may lead to the ocean being reducing or oxidizing. If it is more reducing than the Earth’s ocean, then CH${}_{4}$, rather than CO${}_{2}$, would be the main carbon compound outgassed by potential hydrothermal vents which would inhibit methanogenesis [185]. On the other hand, the production of H${}_{2}$ by serpentinization may tend to counter this trend and lead to feedstock for methanogens. However, due to the fact that the icy satellites likely accreted from a mixture of ice and rock, the subsurface water reservoirs should be rather oxidizing [186]. In addition, the salinity of the aquifers might vary. Estimates for subsurface oceans range from a few tens of grams of salt per kilogram water to scenarios of liquids saturated with MgSO${}_{4}$ or NaCl. Methanogens are known to resist salinities of up to 1.71–2.57 mol·L${}^{-1}$ NaCl [187]. Moreover, potential subsurface aquifers may consist of a non negligible amount of ammonia (NH${}_{3}$). This could lower the freezing temperature of the aquifer but on the other hand it could act as an inhibitor for potential life, if concentrations are too high. Hussmann *et al.* stated that an NH${}_{3}$-concentration of up to 33%, *i.e.*, 14.9 mol·L${}^{-1}$ NH${}_{4}$, seems geochemically plausible. For Europa’s subsurface ocean a range of 2.1%–24.4% NH${}_{3}$ was calculated depending on the assumptions about its interior structure [188]. Such a high ammonia concentration would definitely lead to a serious damage of amino acids [189]. A further important parameter is the time scale in which the aquifers have stayed liquid—at least 10${}^{5}$ years, *i.e.*, geological timescales, are necessary in order for an aquifer to become a habitat for any life form [190].

In the following subsection we will describe several icy moons (for characteristic parameters see Table 3) and their potential to host methanogens.

#### 3.3.1. Enceladus

Enceladus is a rather small icy moon that orbits Saturn at a mean distance of approximately 238 042 km [191]. It has a radius of about 252.1 km and a mass of 1.08$\cdot 10^{20}$ kg [151]. Enceladus has a mean density of about 1 609 ± 5 kg m${}^{-3}$ [160]. This is a rather high value for a small icy moon which strengthens the hypothesis that Enceladus contains a huge amount (about 50%) of rocky material [163]. If Enceladus is differentiated, which reflects the current state of knowledge, it might be separated into a rocky core and an icy shell. The icy shell likely contains ”misty ice caverns“ [192] and/or a (global) subsurface ocean in contact with the rocky core [163,193,194]. According to several models, the rocky core expands to a radius of about 160 to 190 km [193,195,196].

In 2004, the NASA spacecraft Cassini went into orbit around Saturn and since then it has been flown past Titan, Enceladus, and various other moons for numerous times. At least since the third close flyby around Enceladus in July 2005 there has been no doubt about the evidence of a plume of emerging water vapour and small icy grains from warm fractures near the south polar region (see, e.g., [162]). Since the detection of the plume, there has been a debate about its origin—two main points of view emerged: On the one hand, the idea that the plume’s source is the decomposition of ice (sublimating ice) and on the other hand that the plume originates in a subsurface liquid source like caverns, a sea or even a global subsurface ocean. The latter theory was strengthened by new Cassini gravity data received in 2014 [163]. The authors estimate Enceladus’ local subsurface sea to be situated under a 30 to 40 km thick ice shell and extending from the south pole to about 50${}^{\circ }$ south latitude. Another indication for a subsurface aquifer was the detection of E-ring grains that are rich in sodium salts, which can only be formed in liquid water [194,197,198]. Just recently, Thomas *et al.* presented new findings about the physical libration of Enceladus, which point to a global ocean on Enceladus [199].

According to Cassini’s INMS (Ion and Neutral Mass Spectrometer), the main components of Enceladus’ plume are water (87%, volume mixing ratio), H${}_{2}$ (11%), CO${}_{2}$ (0.52%), N${}_{2}$ (less than 0.61%), small amounts of organics (e.g., CH${}_{4}$ and formaldehyde (H${}_{2}$CO)), hydrogen sulfide (H${}_{2}$S), and ${}^{40}$Ar (for detailed composition, see [200,201]). In contrast to previous studies, Waite *et al.* were able to detect NH${}_{3}$ for the first time [201]. Ammonia (coupled with the presence of CH${}_{3}$OH and/or salts) acts as an antifreeze which can reduce the freezing point of the aquifer. The authors concluded that the presence of NH${}_{3}$ combined with the detection of Na${}^{+}$- and K${}^{+}$-salts in the E-ring (second outermost ring of the Saturnian ring system) ice particles is a strong indication for a liquid water reservoir in the interior of Enceladus.

There exist several problems in deducing the subsurface composition by plume observations. For example, the Cassini instrument can only detect neutral atoms and molecules and positive ions [197], which makes, e.g., chlorine ions (*Cl${}^{-}$*) invisible for the detector. So anions can only be detected indirectly. Moreover, the closed ion source is used for most neutrals, but does not work for reactive neutrals such as salts, which must be detected by the open ion source at a much reduced sensitivity. Furthermore, it has to be considered that there may be far more types of complex molecules in the subsurface reservoir which do not find their way to the surface. Hence, Cassini’s INMS was only able to detect relatively light organics due to the lack of proper instruments- just up to a molecular weight of 100 atomic mass units. This means, that if there are biologically relevant molecules like amino acids, nucleotides, or proteins in the plume material, Cassini has not been able to detect them.

Nevertheless, analysing the plume particles is the main starting point to estimate the chemical composition of the potential subsurface water aquifer. For example, Hsu *et al.* analysed the silicon-rich, nanometre-sized dust particles and reported an indirect proof for the existence of hydrothermal activities within Enceladus [202]. In the context of methanogenic life, it is interesting to note that CH${}_{4}$ was part of the plume composition and its existence under conditions that would favour methane clathrate formation in the ocean has been argued as another indicator of hydrothermal activity [200]. If CH${}_{4}$ is produced biologically, than this will likely take place in the subsurface aquifer. Further observations regarding the isotopic composition of this CH${}_{4}$ would give an indication about the origin of the molecules (see Section 3.1).

However, the habitability of Enceladus is still strongly debated due to the high heat flux and loss rate from the plumes. However, recent reanalysis of Cassini’s CIRS (Cassini Composite Infrared Spectrometer) data by Howett *et el.* suggest that the heat flux (4.64±0.23 GW, [203]) is significantly lower than previously estimated (15.8±3.1 GW, [204]). Nevertheless, the high loss rate from the plume indicates that the plumes and Enceladus’ potential subsurface aquifer are temporary events. Hansen *et al.* reported the rate of water vapour injection to be 200 kg s${}^{-1}$ [205] (with a standard deviation of 30 kg s${}^{-1}$)—this would lead to a loss of 20% Enceladus mass if the current plume gas production rate had been constant over the age of the Solar System. It is assumed that Enceladus’ plume is erupting and feeding Saturn’s E-ring at an approximately constant rate at least for the last 300 years [206]. According to several numerical studies of the ring particle dynamics the lifetime of the ring particles is less than 200 years which implies that some kind of resupplying process, *i.e.*, permanent production of Enceladus’ plume particles, must exist to keep the E-ring stable [207].

**Figure 5:**
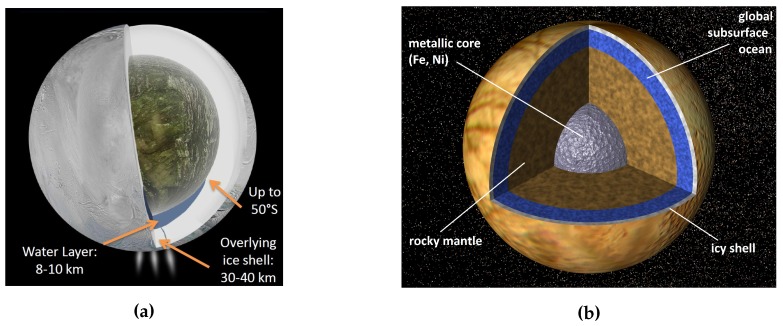
Artist’s concepts of the potential interior structure of Enceladus and Europa. (**a**) Potential interior of Enceladus according to [163], Image credit: NASA/JPL-Caltech (PIA18071), adapted; (**b**) Potential interior of Europa according to [208], Image credit: NASA/JPL (PIA01130), adapted.

#### 3.3.2. Europa

Before Cassini detected Enceladus’ plumes, Europa had been by far the most promising object in the outer Solar System concerning the possibility of being a habitat for putative extraterrestrial life. In a typical two-layer model of Europa assuming a moment of inertia factor (MoI) of 0.346 and the mean density of the outer shell to be about 1000 kg m${}^{-3}$, the core radius is about 1400 km and has a density of about 3800 kg m${}^{-3}$ [209]. This results in a low density outer water shell with a thickness of about 80–170 km [186], depending on the assumed parameters and models. The hydrostatic pressure at the base of this water layer is about 84–180 MPa (according to typical three-layer models presented in [208,209]). Even if the pressure might be too high on the ocean’s floor, methanogens could resist easily the pressure at a depth of approx. 30 km which would be comparable to the hydrostatic pressure at the average terrestrial seafloor depth of 4 km [190]. A main factor for the habitability of Europa’s ocean will be active material cycles that transport reductants from the ocean floor and oxidants from the overlying ice shell to the habitable zone inside the aquifer [190].

NASA’s Galileo mission launched in 1989 revealed Europa to have a subglacial liquid water layer beneath a solid icy crust [210]. The thickness of the ice shell and the liquid water layer is still highly debated due to the lack of accurate gravimetric and altimetric measurements. By now, there are two models proposed, that describe the dimensions of the liquid to the solid water shells in a contrasting way: the “thin” (e.g., [211,212]) and “thick” model. The first model implies, that the icy layer may be less than 10 km thick which means that there might be a link between the underlying ocean and the surface, e.g., through cracks. The “thick” model predicts an icy shell of about 20 km. It seems rather likely that the truth lies in a combination of these two models with a global variable ice shell thickness because none of these models can explain every single detection sufficiently. Notably, the amount of subsurface liquid water on Europa exceeds the water in Earth’s oceans 2–3 times by volume [213].

Knowledge about the topography of Europa (*i.e.*, the existence of hydrothermal vents, volcanoes, and ridges) will remain hidden without future missions like the recently approved Europa multiple flyby missions of ESA (JUICE, launch planned for 2022) or NASA (Europa mission, launch in the 2020’s) to this icy moon. Currently is is assumed that the subsurface ocean is most likely in direct contact with Europa’s silicate core [190]. If so, these circumstances would have led to a geochemically rich environment with all necessary requirements for the existence of methanogens and life in general.

While there is some information about the potential composition of Enceladus’ subsurface water reservoir due to the composition of the plume particles, the composition of Europa’s ocean remains hidden. Though, there was an announcement of the discovery of plumes on Europa in 2012 [214], it was not possible to confirm these detections since then or even in older data. Further detections and subsequent analysis of the potential Europa plume particles would definitely help us to understand the interior ocean conditions at Europa. If we assume (chemical) exchange processes (e.g., serpentinization [215]) between the subsurface ocean and Europa’s surface, one can draw inferences from the surface material about the composition of the aquifer. For example, the detection of CO${}_{2}$ by NASA Galileo mission in the hydrate-rich terrains on Europa’s surface gives a hint to the oxidizing state of the subsurface aquifer [186]. There might even be the chance that potential biomass would be transported to the surface, but the concentration would be extremely small which would make biosignatures hard to detect (about one cell per mL [190]). However, the conditions at the surface are extremely different from subsurface, *i.e.*, the particles undergo changes due to abrupt variation in temperature and the exposure to the high Jovian radiation. Nevertheless, there are several studies about its potential chemical composition (see, e.g., [215])

McCollom analysed the possibility of methanogenesis to occur for two different scenarios for a hydrothermally active Europa [216]. He considered a relatively reduced ocean (dominated by CH${}_{4}$ and H${}_{2}$S) and a relatively oxidized ocean (dominated by SO${}_{4}^{2-}$ and HCO${}_{3}^{-}$) [216]. The author estimated a biomass productivity on Europa of 10${}^{5}$ to 10${}^{6}$ kg yr${}^{-1}$, which is equal to 10 ppm of the hydrothermal biomass productivity on Earth [190].

#### 3.3.3. Titan

Titan is unique among the Solar System satellites due to its dense atmosphere (1.5 bar) which just above the surface consists mainly of N${}_{2}$ (approx. 94.5% mole fraction), CH${}_{4}$ (5.5%), and various trace constituents like H${}_{2}$, ethane (C${}_{2}$H${}_{6}$) and hydrogen cyanide (HCN) [217,218,219] and because it is the only other Solar System object besides the Earth with standing entities of some liquid at its surface. Its atmosphere extends to an altitude of 1 500 km [220]. Although much colder (mean surface temperature of 94 K, see Table 3), there are some similarities to the Earth like the qualitatively similar vertical structure of the atmosphere, the presence of a complex liquid solution cycle (on Titan CH${}_{4}$ plays the role of water since the temperature and pressure conditions put methane at its triple point at the surface), or the active organic chemistry [218]. Titan is often quoted as a cold analogue for the prebiotic Earth [189,221,222,223,224]. There might be the possibility that liquid water (or at least liquid water-ammonia mixture) existed even at the surface caused by impacts of, e.g., comets that could melt surface water ice or due to cryovolcanism [225] for geological short periods of time (100 to 1 000 years assumed for a crater with 15km diameter, 1000 to 10 000 years for the ones with 150km [226]). Independent of these considerations, the average Titan surface is in fact too cold and not energetic enough to serve as a habitat for life-as-we-know-it [218].

**Figure 6:**
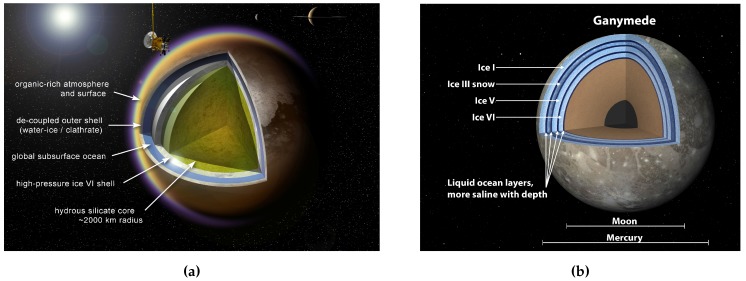
Artist’s concepts of the potential interior structure of Titan and Ganymede. (**a**) Potential interior of Titan according to [229], Image credit: A. D. Fortes/UCL/STFC (PIA14445); (**b**) Potential interior of Ganymede according to [166], Image credit: NASA/JPL-Caltech (PIA18005).

However, Titan likely harbors a liquid water(-ammonia) ocean a few hundred kilometres thick under an ice shell of about 100 km [227,228]. In contrast to Europa or Enceladus, the ocean does not seem to be in contact with the rocky mantle/core but with an icy ocean floor (see Figure 6a) which makes life in this aquifer rather unlikely [228].

#### 3.3.4. Other Icy Moons

In the following, we will present several other icy moons where a subsurface liquid water layer seems to be possible due to models and corresponding observations. However, these satellites are either unlikely to harbor life-as-we-know-it (Ganymede, Mimas, Dione, Callisto) or there are not enough data available to draw conclusions about their possible interior structure (Triton, Rhea, Oberon, Titania).

##### Ganymede

Ganymede is the largest satellite in the Solar System. Its interior seems to consist of several layers of different ice phases (due to the increasing pressure at increasing depth) separated by liquid water layers (see Figure 6b). The lowest liquid layer may be in contact with the rocky mantle of Ganymede. Due to the high pressure at this depth (about 800 km), the temperature is higher than in the upper layers [166]. However, an estimated pressure of approx. 500 MPa at the ocean floor is possibly too high for life-as-we-know-it [209].

##### Mimas and Dione

Dione has the third highest density of the Saturnian satellites which suggests that it contains a large amount of dense material like silicates. Cassini’s magnetometer detected hints of a faint plume of charged particles and bright, fresh fractures on its surface. On the other hand, Cassini’s Visual Infrared Mapping Spectrometer (VIMS) was not able to detect any evidence for plumes [230]. According to the authors activity must be below our threshold of detection, or it could be occurring but was not yet captured on the observations at large solar phase angles [230]. However, current geologic activity or a subsurface water reservoir cannot absolutely be ruled out [231].

Mimas is another icy moon of the Saturnian system. A study published in 2014 shows that Mimas “has either a large nonhydrostatic interior, or a hydrostatic one with an internal ocean beneath a thick icy shell” [232].

There is currently too little information about these icy satellites available in order to make justifiable statements on the potential habitability of these bodies. Furthermore, the study of Hussmann *et al.* exclude Mimas and Dione (as well as Enceladus and Tethys) from possessing a subsurface water layer [188].

##### Callisto

It is rather likely that Callisto harbours a subsurface ocean, but due to the fact that its interior is most likely not fully differentiated, this aquifer should not be habitable for life-as-we-know-it.

##### Triton

The largest satellite of Neptune is special among the large moons in the outer Solar System due to its retrograde orbit. Triton is most likely a captured Kuiper belt object (KBO) [233] and its dimensions, density, temperature and chemical composition is similar to those of Pluto (see Table 3). Triton is most likely an active moon as Voyager 2 observed dark geyser-like eruptions in the summer of 1989 [234]. Although the surface of Triton is very harmful to life due to the fact that this moon lies within the magnetosphere of Neptune, there is still the possibility that the putative subsurface ocean may harbour life. On the other hand, the ocean may be ammonia-rich and there may be a limited abundance of carbon both of which would argue against life on Triton [233].

##### Rhea, Oberon, Titania

Saturn’s second largest satellite Rhea and Uranus’ largest moons Oberon and Titania may possess a liquid water layer [188]. According to a study by Hussmann *et al.*, Oberon may have a liquid layer of up to 40 km, Titania of 52 km, and Rhea of 17 km [188]. Rhea’s layer may be relatively thin compared to its icy layer of about 400 km. However, these assumptions are just theoretical and have not been verified by observations yet (see [231]).

#### 3.3.5. Dwarf Planets and Small Solar System Bodies

Currently, the International Astronomical Union (IAU) categorizes 5 bodies as dwarf planets: Ceres, Pluto, Haumea, Makemake, and Eris. A dwarf planet, as defined in the IAU Resolution B5, is “a celestial body that (a) is in orbit around the Sun; (b) has sufficient mass for its self-gravity to overcome rigid body forces so that it assumes a hydrostatic equilibrium (nearly round) shape; (c) has not cleared the neighbourhood around its orbit; and (d) is not a satellite. All other objects, except satellites, orbiting the Sun shall be referred to collectively as ‘Small Solar System Bodies’ ” [235]. The latter includes small Trans-Neptunian Objects (TNOs), asteroids, comets, *etc.* According to the study of Hussmann *et al.*, subsurface oceans are possible on the dwarf planets Pluto and Eris, as well as on the TNOs Sedna and 90482 Orcus [188].

Furthermore, Ceres, the largest object in the asteroid belt with an effective radius of 476.2 ± 1.7 km [150], may host a subsurface water reservoir, too. In January 2014, emissions of water vapour were detected by using ESA’s Herschel space observatory [236]. Observations by NASA’s Dawn spacecraft showed that the reason for the detected bright spots are more likely vaporization or sublimation of ice than cryovolcanism [237]. On the course of this mission new insights will be gained into the internal structure of this dwarf planet.

Another highlight in 2015 for solar system exploration was the NASA’s New Horizons spacecraft flyby of Pluto (effective radius 1195 ± 5 km [150]) and its satellites. Based on measurements of Pluto’s shape, we will receive better knowledge about the interior structure of Pluto and if this dwarf planet may harbour a subsurface aquifer [238]. Even Pluto’s largest moon Charon (effective radius 605 ± 8 km [150]) might have possessed a subsurface aquifer in its past [239].

As can be seen from these examples, subsurface aquifers may be more frequent in small Solar System bodies than usually thought. Nevertheless, life on small bodies like TNOs is rather unlikely due to their huge distance to the Sun. The temperature is extremely low, the necessary supply of energy, e.g., tidal energy, is not clear in this context as it is for the Galilean satellites and at Enceladus. Furthermore, the availability of the necessary elements seems rather unlikely due to the history of these objects. TNOs are remnants of the early outer Solar System and therefore their average composition is mostly comet-like [240]. This means that they contain just a small amount of rocky material and hence may not host an adequate amount of chemical compounds essential for life.

## 4. Simulating Extraterrestrial Conditions

Over the last decades, a plethora of simulations under Mars-like conditions were performed with microorganisms. Due to the detection of CH${}_{4}$ in the Martian atmosphere (see Section 3.2) or in Enceladus’ plume particles [2] and due to the ability of methanogens to withstand extreme conditions (see Section 2), methanogens were emphasized as model organisms in Astrobiology. In the following, we will present a selection of various astrobiological experiments.

### 4.1. Mars-Like Conditions

Investigations regarding growth and survival of *Methanothermobacter wolfeii*, *Methanosarcina barkeri*, *Methanococcus maripaludis* and *Methanobacterium formicicum* under Martian-like conditions have been performed in the Pegasus planetary simulation chamber [58,59]. All methanogens tested survived 60 days at 600 Pa, a pressure found at the Martian surface/subsurface boundary [58]. Moreover, a mixed culture of methanogens has been tested under low-pressure conditions in the Pegasus planetary simulation chamber including desiccation and Martian regolith analogues. *M. wolfeii* and *M. formicicum* did only survive desiccation in a few experiments, whereas *M. barkeri* survived in most cases when incubated with different Martian soil analogues. *M. formicicium* survived only one of the desiccation experiments [59]. Another study by Mickol and Kral showed that the four tested strains were not affected by pressure values of 3.3 kPa and 6.7 kPa, respectively [60]. Furthermore, it was shown (e.g., in [61,62]) that the tested methanogens were able to propagate in the presence of the Martian-like soils *Mojave Mars Simulant* [63] and *JSC Mars-1* [241]. In addition, *M. maripaludis* was tested concerning its growth in the presence of montmorillonite (smectite clay mineral) [62] and (magnesium) perchlorate [64,65]. In both cases, significant amounts of CH${}_{4}$ were still produced. It is important to note, that these experiments require liquid water and a protection from UV radiation present on Mars’ surface, which would be possible in the potential subsurface water reservoirs. Chastain *et al.* published a study that determined “whether biological methanogenesis could occur in non-reduced, non-buffered environments containing only H${}_{2}$, CO${}_{2}$, montmorillonite, and the liquid fraction extracted from a montmorillonite/deionized water suspension” [66]. The authors concluded that montmorillonite can supply micronutrients to the potential environmental system essential for methanogenesis. However, a mixture of montmorillonite and zero-valent iron would be even better [242]. On Mars, where ferrous minerals are abundant, zero-valent iron could be an alternative energy source for methanogens [67].

Other studies investigated the ability of methanogens to withstand freeze-thaw-cycles [243,244]. Djordjevic *et al.* tested the ability of *M. wolfeii*, *M. barkeri*, *M. maripaludis* and *M. formicicum* to survive short-term freeze/thaw cycles (approx. 20–50 days) between temperatures of −80${}^{\circ }$C and +37${}^{\circ }$C [243], whereas Mickol *et al.* tested *M. wolfeii* and *M. formicicum* in a longer period (about 281 days) in a temperature range of −80${}^{\circ }$C and +55${}^{\circ }$C [244]. In both studies, at least some of the strains were able to withstand prolonged low-temperature conditions. A further study tested the survivability of methanogens under Martian thermal conditions [68]. Here, six strains of methanogens [245] were tested for “diurnal temperature fluctuations in the range from −75 to +20 ${}^{\circ }$C and humidity fluctuations between a${}_{w}$-values of 0.1 and 0.9 in a Mars-like atmosphere dominated by CO${}_{2}$ (95.3%)” [68]. This three weeks lasting study showed that methanogens found in the terrestrial permafrost have an unexpectedly high survivability rate under Mars-like thermal conditions. In the meantime, using laser spectroscopy Schirmack *et al.* were able to show the growth of *M. soligelidi* under Martian thermo-physical conditions continuously during the experiment and not just through comparison of the number of cells and CH${}_{4}$ production rate at the beginning and at the end of the experiment [69].

The above mentioned experiments consolidated the role of methanogens to be a hot candidate for life on Mars.

The upcoming ExoMars mission, which consists of a Trace Gas Orbiter plus an entry, descent and landing demonstrator module as well as a rover and a surface platform (planned launches in 2016 and 2018, respectively) will focus on the search for biosignatures of Martian life. The rover will have a Raman spectrometer on board. Current studies describe how this instrument may be used to characterize potential methanogenic archaea, based on experiments with *M. soligelidi* on two different Martian-like soils [70,71].

### 4.2. Icy Moon-Like Conditions

If a subsurface ocean/water reservoir on an icy moon hosted life, the organisms would have to be chemolithoautotroph and anaerobic. In their paper, McKay *et al.* describe the fact that there are only three known microbial ecosystems on Earth which meet these requirements and, therefore, could exist on icy moons [246]. Two of these systems “are based on methanogens [247] that use H${}_{2}$ derived from rock-water reactions [248,249] and a third on sulfur-reducing bacteria [250] that use redox couples produced ultimately by radioactive decay [251]” [246]. The latter process takes place at high temperature which might be a limiting factor for this metabolism on icy moons. Therefore, mainly psychrophilic methanogens are likely to be the only known terrestrial microorganisms that could thrive on an icy moon. For Enceladus, several of the compounds detected by Cassini can serve as substrates (e.g., H${}_{2}$, N${}_{2}$, or CH${}_{3}$OH) for them or/and may even be products (e.g., CO${}_{2}$, CO, or CH${}_{4}$) of the methanogenic metabolism.

Several species of methanogens have already been tested in connection to icy moons (e.g., [252]), but none of these experiments were done under cold, general icy-moon like subsurface aquifer conditions, this means for example at temperatures of less than 20 ${}^{\circ }$C (locally, the temperature can be higher, especially near hydrothermal vents [202]). Additionally, experiments aimed to determine the resistance of certain methanogens against a high amount of ammonia are also missing. Studies with ammonium chloride (NH${}_{4}$Cl) with concentration up to 6%, *i.e.*, 0.272 mol·L${}^{-1}$, were performed [253]. The authors report that *M. wolfeii* show CH${}_{4}$ production on concentrations of ammonium chloride as high as 4%, whereas *M. maripaludis* seems to be resistant against 3% NH${}_{4}$Cl. These experiments are very important because there might be a certain amount of ammonia in the subsurface liquid that could act as an antifreezing agent (see Section 3.3). Furthermore, there are running studies that deal with the possibility to cultivate methanogens under Enceladus-like conditions [254]. These include the composition of the medium, the gas phase, the temperature range, and the pressure conditions in the subsurface aquifer.

### 4.3. Terrestrial Analogues for Solar System Objects

There exist no perfect terrestrial analogues that would mimic an ecosystem on neither Mars nor any other Solar System object. However, several places on Earth seem to be at least similar to extraterrestrial locations. Preston and Dartnell present 33 analogous sites for Venus, Mars, Europa, Enceladus, and Titan, which reflect the mineralogical, geochemical, physical, and/or chemical conditions on these bodies [255]. From this list, we want to highlight the Bockfjord Volcanic Complex [256] (Svalbard, Norway) which serves as an analogue for Hesperian Mars [256], the Lidy Hot Springs [249] and the Columbia River Basalts [248] (both USA), which should both roughly represent the conditions on the sea floor of a potential subsurface water reservoir on icy moons. Another terrestrial Mars analogue is the permafrost in the Imuruk lake volcanic field area (Alaska, USA) [257]. In each of these ecosystem, methanogenic activity was detected.

For icy moons, important terrestrial analogues are the subglacial lakes in Antarctica, e.g., Lake Vostok. These ecosystems mirror the cold and dark environmental condition in subsurface aquifers in the best possible way. Ecosystems in such an environment may have been isolated from the outside for more than 15 million years [258]. So far, direct sampling of the liquid water of these systems proves to be rather difficult for similar reasons as for their extraterrestrial locations. For example, drilling through hundreds of meters to several kilometres thick ice shell is required whereby the formed drill hole has to be saved from freezing. On the other hand, the subglacial ecosystem has to be protected against contamination. On icy moons there are several further aggravating aspects that have to be kept in mind at the planning phase of such a drilling mission, e.g., the source of energy.

## 5. Conclusions

### 5.1. Discussion

Methanogens represent one of the most widespread group of microorganisms that span habitats from hot vents in the deep oceans to ice cold permafrost soils and a temperature range between below 0 and up to 122 ${}^{\circ }$C. Their energy metabolism, which is independent of oxygen and often independent of the presence of any organic molecule, is unique and potentially developed early on Earth [19]. Moreover, many methanogens are known to be prototrophs (*i.e.*, microorganism that can synthesize its nutrients/growth factors from inorganic material [259,260]) and as such they are ideal candidates for inhabiting or surviving on planetary bodies other than Earth.

Only a limited number of inconclusive studies on the effect of UV light on methanogens is available in the literature. Few studies focussed on the response on the morphological, physiological and genetic level as a results of applying multiple stress factors in parallel. Analysing a multiple stress response would better enable deducing survival and ecophysiological characteristics of methanogens from laboratory experiments and inferences on a putative comparable situations on other celestial bodies.

Some of the physiological adaptations of methanogens, like “uncoupling”, and their ability to regain a fully active metabolic potential after recovering from dormancy are intriguing features of these organisms. These demonstrate the versatility of methanogens in adapting to changing environmental conditions (e.g., temperature, pH). We hypothesize that uncoupling is a methanogenic ecophysiological adaptation feature not only to redirect the carbon flux from biomass formation, propagation and homoeostasis to energy generation just enabling cell survival, but also to be able to quickly reactivate to full metabolic potential from a state of dormancy. Under special environmental conditions, which can be found at the diffuse vent systems located next to hydrothermal vents on the sea-floor, methanogens are exposed to a narrow temperature gradient (e.g., from >20 ${}^{\circ }$C to 190 ${}^{\circ }$C within 12 cm) in which they might be physiologically active [19,79]. Hence, methanogens might be able to rapidly respond to environmental changes through ecophysiological adaptations, as they are putatively also encountered on other bodies throughout the Solar System.

The strain and species-specific characteristics (specific growth rate, (cell) specific CH${}_{4}$ productivity, Y${}_{\left(x/CH4\right)}$) of methanogens (e.g., [13,78]) have not been considered much regarding their ecophysiological role in the context of astrobiology. However, the characteristics of methanogens to vary, e.g., Y${}_{\left(x/CH4\right)}$) have been successfully employed in biotechnology when the organisms were utilized for the purpose of biological methanation [44,261]. These ecophysiological characteristics could eventually not only be varied during application of certain bioprocess technological conditions, but also be intriguing to study in the context of astrobiological research, when environmental constraints are forcing the methanogens into maintaining energy homoeostasis until the organisms are capable to propagate.

### 5.2. Outlook

The different adaptations and facets of methanogens make these organisms and their metabolism important study objects in the context of search for life on other planets and solar bodies as they help to define prerequisites and physical limits for life. Taken together recent data and missions to the moons of the outer Solar System have revealed a number of additional celestial bodies that could potentially have hosted an evolution of life independent (or parallel) to that of Earth.

In the future, research should focus on missions to other Solar System bodies that will not just identify potential habitats but signs for life with the aid of instruments adjusted to the respective environment. For example, the upcoming ExoMars rover will focus on the search for biosignatures of life on Mars. The rover will have a Raman spectrometer on board that can be used to characterize potential methanogenic archaea, based on experiments with *M. soligelidi* on two different Martian-like soils [70,71]. ESA’s Jupiter Icy Moon Explorer (JUICE) is planned to start in 2022 and will arrive at the Jovian system most likely in 2030 [262]. The focus of this mission lies on Ganymede as a planetary body and potential habitat, but it will also do investigations of Callisto and Europa [263]. For the planned NASA Europa flyby mission, the focus will lie on the chemistry essential to life, including organic molecules, and it will search for characteristic surface features that could give a hint for future missions [264]. Both JUICE and the NASA Europa mission will have a spectrometer on board that will be able to detect molecules with a higher molecular mass than the compounds found with Cassini’s INMS in Enceladus’ plume. Hence, if there are plumes on Europa which are fed by the subsurface water reservoir, this spectrometer might be able to detect direct signs of life-as-we-know-it, like nucleotides, proteins, or even cells. *In situ* measurement with convenient instruments at the plumes of Europa (as already mentioned, the plumes on Europa are still not confirmed) or Enceladus will be a major step towards a better understanding of the potential of life in the Solar System. In the distant future, missions containing cryobots (*i.e.*, probes that will be able to penetrate water ice) might provide an opportunity to perform *in situ* measurements within the subsurface aquifer. In this context, e.g., NASA in cooperation with Stone Aerospace is already working on such robots. The next step for this project named VALKYRIE (Very-Deep Autonomous Laser-Powered Kilowatt-Class Yo-Yoing Robotic Ice Explorer) will be the testing of a small version of VALKYRIE during three field campaigns in Canada and Norway [265]. Nevertheless, to adapt this technical system to the prevailing conditions on Europa or Enceladus will be a major issue. At the moment, the exact thickness of the ice shell of these bodies is still not precisely known. It makes a great difference to drill through some hundreds of meters or 30 kilometres of ice. Furthermore, the energy source and the way of communication between the different instruments (at least between the cryobot, a landing probe, an orbiter, and instruments on Earth) remains still an unsolved problem.

The potential of methanogens for astrobiological studies is still a long way from being exhausted. Better understanding of the Solar System bodies will give us the chance to improve the experiments. Therefore, we would recommend a interdisciplinary collaboration among the various scientific fields of astrobiology to move closer to the ultimate goal: the detection of extraterrestrial life.

## References

[1] Niemann H.B., Atreya S.K., Bauer S.J., Carignan G.R., Demick J.E., Frost R.L., Gautier D., Haberman J.A., Harpold D.N., Hunten D.M. (2005). The abundances of constituents of Titan’s atmosphere from the GCMS instrument on the Huygens probe. Nature.

[2] Waite J.H., Brockwell T., Lewis W.S., Magee B., McKinnon W.B., Mousis O., Bouquet A. (2014). Enceladus Plume Composition. LPI Contrib..

[3] Formisano V., Atreya S., Encrenaz T., Ignatiev N., Giuranna M. (2004). Detection of Methane in the Atmosphere of Mars. Science.

[4] Mumma M.J., Villanueva G.L., Novak R.E., Hewagama T., Bonev B.P., DiSanti M.A., Mandell A.M., Smith M.D. (2009). Strong Release of Methane on Mars in Northern Summer 2003. Science.

[5] Webster C.R., Mahaffy P.R., Atreya S.K., Flesch G.J., Mischna M.A., Meslin P.Y., Farley K.A., Conrad P.G., Christensen L.E., Pavlov A.A. (2015). Mars methane detection and variability at Gale crater. Science.

[6] Liu Y., Whitman W.B. (2008). Metabolic, Phylogenetic, and Ecological Diversity of the Methanogenic Archaea. Ann. N. Y. Acad. Sci..

[B7-life-05-01652] 7.There are also (aerobic) marine microorganisms known to produce CH_4_ from methylphosphonic acid [266,267,268].

[8] Offre P., Spang A., Schleper C. (2013). Archaea in Biogeochemical Cycles. Annu. Rev. Microbiol..

[9] Evans P.N., Parks D.H., Chadwick G.L., Robbins S.J., Orphan V.J., Golding S.D., Tyson G.W. (2015). Methane metabolism in the archaeal phylum Bathyarchaeota revealed by genome-centric metagenomics. Science.

[10] Thauer R.K., Kaster A.K., Seedorf H., Buckel W., Hedderich R. (2008). Methanogenic archaea: Ecologically relevant differences in energy conservation. Nat. Rev. Microbiol..

[11] Borrel G., O’Toole P.W., Harris H.M.B., Peyret P., Brugère J.F., Gribaldo S. (2013). Phylogenomic Data Support a Seventh Order of Methylotrophic Methanogens and Provide Insights into the Evolution of Methanogenesis. Genome Biol. Evol..

[12] Iino T., Tamaki H., Tamazawa S., Ueno Y., Ohkuma M., Suzuki K.i., Igarashi Y., Haruta S. (2013). Candidatus Methanogranum caenicola: A Novel Methanogen from the Anaerobic Digested Sludge, and Proposal of Methanomassiliicoccaceae fam. nov. and Methanomassiliicoccales ord. nov., for a Methanogenic Lineage of the Class Thermoplasmata. Microbes Environ..

[13] Rittmann S., Seifert A., Herwig C. (2015). Essential prerequisites for successful bioprocess development of biological CH_4_ production from CO_2_ and H_2_. Crit. Rev. Biotechnol..

[14] Ferry J.G. (2010). How to make a living by exhaling methane. Annu. Rev. Microbiol..

[15] Oelgeschläger E., Rother M. (2009). Influence of carbon monoxide on metabolite formation in Methanosarcina acetivorans. FEMS Microbiol. Lett..

[16] Rother M., Metcalf W.W. (2004). Anaerobic growth of Methanosarcina acetivorans C2A on carbon monoxide: An unusual way of life for a methanogenic archaeon. Proc. Natl. Acad. Sci. USA.

[17] Brocks J.J., Logan G.A., Buick R., Summons R.E. (1999). Archean Molecular Fossils and the Early Rise of Eukaryotes. Science.

[18] Ueno Y., Yamada K., Yoshida N., Maruyama S., Isozak Y. (2006). Evidence from fluid inclusions for microbial methanogenesis in the early Archaean era. Nature.

[19] Martin W., Baross J., Kelley D., Russell M.J. (2008). Hydrothermal vents and the origin of life. Nat. Rev. Microbiol..

[20] Brochier-Armanet C., Forterre P., Gribaldo S. (2011). Phylogeny and evolution of the Archaea: One hundred genomes later. Curr. Opin. Microbiol..

[21] Blank C.E. (2009). Phylogenomic dating—The relative antiquity of archaeal metabolic and physiological traits. Astrobiology.

[22] Nakamura K., Takahashi A., Mori C., Tamaki H., Mochimaru H., Nakamura K., Takamizawa K., Kamagata Y. (2013). Methanothermobacter tenebrarum sp. nov., a hydrogenotrophic, thermophilic methanogen isolated from gas-associated formation water of a natural gas field. Int. J. Syst. Evol. Microbiol..

[23] Ma K., Liu X., Dong X. (2006). Methanosaeta harundinacea sp. nov., a novel acetate-scavenging methanogen isolated from a UASB reactor. Int. J. Syst. Evol. Microbiol..

[24] Lü Z., Lu Y. (2012). *Methanocella conradii* sp. nov., a Thermophilic, Obligate Hydrogenotrophic Methanogen, Isolated from Chinese Rice Field Soil. PLoS ONE.

[25] L’Haridon S., Reysenbach A.L., Banta A., Messner P., Schumann P., Stackebrandt E., Jeanthon C. (2003). Methanocaldococcus indicus sp. nov., a novel hyperthermophilic methanogen isolated from the Central Indian Ridge. Int. J. Syst. Evol. Microbiol..

[26] Jones W.J., Leigh J.A., Mayer F., Woese C.R., Wolfe R.S. (1983). *Methanococcus jannaschii* sp. nov., an extremely thermophilic methanogen from a submarine hydrothermal vent. Arch. Microbiol..

[27] Jiang B., Parshina S.N., Doesburg W.V., Lomans B.P., Stams A.J.M. (2005). Methanomethylovorans thermophila sp. nov., a thermophilic, methylotrophic methanogen from an anaerobic reactor fed with methanol. Int. J. Syst. Evol. Microbiol..

[28] Jeanthon C., L’Haridon S., Reysenbach A.L., Vernet M., Messner P., Sleytr U.B., Prieur D. (1998). Methanococcus infernus sp. nov., a novel hyperthermophilic lithotrophic methanogen isolated from a deep-sea hydrothermal vent. Int. J. Syst. Bacteriol..

[29] Jeanthon C., L’Haridon S., Reysenbach A.L., Corre E., Vernet M., Messner P., Sleytr U.B., Prieur D. (1999). *Methanococcus vulcanius* sp. nov., a novel hyperthermophilic methanogen isolated from East Pacific Rise, and identification of *Methanococcus* sp. DSM 4213T as *Methanococcus fervens* sp. nov.. Int. J. Syst. Bacteriol..

[30] Cheng L., Qiu T.L., Yin X.B., Wu X.L., Hu G.Q., Deng Y., Zhang H. (2007). Methermicoccus shengliensis gen. nov., sp. nov., a thermophilic, methylotrophic methanogen isolated from oil-production water, and proposal of Methermicoccaceae fam. nov.. Int. J. Syst. Evol. Microbiol..

[31] Parshina S.N., Ermakova A.V., Bomberg M., Detkova E.N. (2014). Methanospirillum stamsii sp. nov., a psychrotolerant, hydrogenotrophic, methanogenic archaeon isolated from an anaerobic expanded granular sludge bed bioreactor operated at low temperature. Int. J. Syst. Evol. Microbiol..

[32] Von Klein D.v., Arab H., Völker H., Thomm M. (2002). Methanosarcina baltica, sp. nov., a novel methanogen isolated from the Gotland Deep of the Baltic Sea. Extremophiles.

[33] Franzmann P.D., Liu Y., Balkwill D.L., Aldrich H.C., Macario E.C.D., Boone D.R. (1997). Methanogenium frigidum sp. nov., a Psychrophilic, H_2_-Using Methanogen from Ace Lake, Antarctica. Int. J. Syst. Bacteriol..

[34] Wagner D., Schirmack J., Ganzert L., Morozova D., Mangelsdorf K. (2013). *Methanosarcina soligelidi* sp. nov., a desiccation- and freeze-thaw-resistant methanogenic archaeon from a Siberian permafrost-affected soil. Int. J. Syst. Evol. Microbiol..

[35] Schirmack J., Mangelsdorf K., Ganzert L., Sand W., Hillebrand-Voiculescu A., Wagner D. (2014). Methanobacterium movilense sp. nov., a hydrogenotrophic, secondary-alcohol-utilizing methanogen from the anoxic sediment of a subsurface lake. Int. J. Syst. Evol. Microbiol..

[36] Mondav R., Woodcroft B.J., Kim E.H., McCalley C.K., Hodgkins S.B., Crill P.M., Chanton J., Hurst G.B., VerBerkmoes N.C., Saleska S.R. (2014). Discovery of a novel methanogen prevalent in thawing permafrost. Nat. Commun..

[37] Rittmann S., Holubar P. (2014). Rapid extraction of total RNA from an anaerobic sludge biocoenosis. Folia Microbiol..

[38] Cavicchioli R. (2006). Cold-adapted archaea. Nat. Rev. Microbiol..

[39] Huber R., Kurr M., Jannasch H.W., Stetter K.O. (1989). A novel group of abyssal methanogenic archaebacteria (Methanopyrus) growing at 110 ^∘^C. Nature.

[40] Rittmann S.K.-M.R., Seifert A., Herwig C. (2012). Quantitative analysis of media dilution rate effects on Methanothermobacter marburgensis grown in continuous culture on H_2_ and CO_2_. Biomass Bioenergy.

[41] Rittmann S. K.-M. R., Guebitz G.M. (2015). A critical assessment of microbiological biogas to biomethane upgrading systems. Biogas Science and Technology.

[42] Seifert A.H., Rittmann S., Bernacchi S., Herwig C. (2013). Method for assessing the impact of emission gasses on physiology and productivity in biological methanogenesis. Bioresour. Technol..

[43] Seifert A.H., Rittmann S., Herwig C. (2014). Analysis of process related factors to increase volumetric productivity and quality of biomethane with Methanothermobacter marburgensis. Appl. Energy.

[44] Bernacchi S., Rittmann S., Seifert A.H., Krajete A., Herwig C. (2014). Experimental methods for screening parameters influencing the growth to product yield (Y_(*x*/*CH*4)_) of a biological methane production (BMP) process performed with Methanothermobacter marburgensis. AIMS Bioeng..

[45] Albers S.V., Meyer B.H. (2011). The archaeal cell envelope. Nat. Rev. Microbiol..

[46] UWr Wydział Biotechnologii (2013). Methanogens Database. http://metanogen.biotech.uni.wroc.pl/.

[47] Leibniz-Institut DSMZ (2015). Catalogue of Microorganisms. http://www.dsmz.de/catalogues/catalogue-microorganisms.html.

[48] Schönheit P., Moll J., Thauer R.K. (1979). Nickel, cobalt, and molybdenum requirement for growth of Methanobacterium thermoautotrophicum. Arch. Microbiol..

[49] Bonacker L.G., Baudner S., Mörschel E., Böcher R., Thauer R.K. (1993). Properties of the two isoenzymes of methyl-coenzyme M reductase in Methanobacterium thermoautotrophicum. Eur. J. Biochem./FEBS.

[50] Wang M., Tomb J.F., Ferry J.G. (2011). Electron transport in acetate-grown Methanosarcina acetivorans. BMC Microbiol..

[51] Kaster A.K., Moll J., Parey K., Thauer R.K. (2011). Coupling of ferredoxin and heterodisulfide reduction via electron bifurcation in hydrogenotrophic methanogenic archaea. Proc. Natl. Acad. Sci. USA.

[52] Huang S.S. (1959). Occurrence of Life Outside the Solar System. Am. Sci..

[53] Kasting J.F., Whitmire D.P., Reynolds R.T. (1993). Habitable Zones around Main Sequence Stars. Icarus.

[54] Leitner J.J., Schwarz R., Firneis M.G., Hitzenberger R., Neubauer D. (2010). Generalizing Habitable Zones in Exoplanetary Systems—The Concept of the Life Supporting Zone. LPI Contrib..

[B55-life-05-01652] 55.The water snow line describes the critical distance from a protostar in a protoplanetary disk where it is cold enough that water condenses into solid ice grains. In the Solar System, this line lies approx. at a distance of 2.7 AU from the Sun [269].

[B56-life-05-01652] 56.The Martian history is roughly divided into three main periods, namely Noachian, Hersperian, and the present period named Amazonian.

[57] Martínez G.M., Renno N.O. (2013). Water and Brines on Mars: Current Evidence and Implications for MSL. Space Sci. Rev..

[58] Kral T.A., Altheide T.S., Lueders A.E., Schuerger A.C. (2011). Low pressure and desiccation effects on methanogens: Implications for life on Mars. Planet. Space Sci..

[59] Kral T.A., Altheide S.T. (2013). Methanogen survival following exposure to desiccation, low pressure and martian regolith analogs. Planet. Space Sci..

[60] Mickol R.L., Kral T.A. Approaching Martian Conditions: Methanogen Survival at Low Pressure. Lunar and Planetary Institute Technical Report, Proceedings of the 45th Lunar and Planetary Science Conference.

[61] Kral T., Bekkum C., McKay C. (2004). Growth of Methanogens on a Mars Soil Simulant. Origin. Life Evol. Biosph..

[62] Mickol R.L., Waddell W.H., Kral T.A. (2014). Methanogens as Models for Life on Mars. LPI Contrib..

[63] Peters G.H., Abbey W., Bearman G.H., Mungas G.S., Smith J.A., Anderson R.C., Douglas S., Beegle L.W. (2008). Mojave Mars simulant-Characterization of a new geologic Mars analog. Icarus.

[64] Kral T.A., Altheide T.S., Lueders A.E., Goodhart T.H., Virden B.T., Birch W., Howe K.L., Gavin P. (2010). Methanogens: A Model for Life on Mars. LPI Contrib..

[65] Goodhart T., Kral T.A. (2010). The Effects of Perchlorate on Methane Production of Methanogens. LPI Contrib..

[66] Chastain B.K., Kral T.A. (2010). Approaching Mars-like Geochemical Conditions in the Laboratory: Omission of Artificial Buffers and Reductants in a Study of Biogenic Methane Production on a Smectite Clay. Astrobiology.

[67] Chastain B.K., Kral T.A. (2010). Zero-valent iron on Mars: An alternative energy source for methanogens. Icarus.

[68] Morozova D., Möhlmann D., Wagner D. (2007). Survival of Methanogenic Archaea from Siberian Permafrost under Simulated Martian Thermal Conditions. Orig. Life Evol. Biosph..

[69] Schirmack J., Böhm M., Brauer C., Löhmannsröben H.G., de Vera J.P., Möhlmann D., Wagner D. (2014). Laser spectroscopic real time measurements of methanogenic activity under simulated Martian subsurface analog conditions. Planet. Space Sci..

[70] Serrano P., Wagner D., Böttger U., de Vera J.P., Lasch P., Hermelink A. (2014). Single-cell analysis of the methanogenic archaeon Methanosarcina soligelidi from Siberian permafrost by means of confocal Raman microspectrocopy for astrobiological research. Planet. Space Sci..

[71] De Vera J.P.P., Böttger U., Fritz J., Weber I., Malaszkiewicz J., Serrano P., Meessen J., Ott S., Wagner D., Hübers H.W. Detection of cyanobacteria and methanogens embedded in Mars analogue minerals by the use of Raman spectroscopy. Proceedings of the 2012 EGU General Assembly Conference.

[72] Archer D.B. (1985). Uncoupling of Methanogenesis from Growth of Methanosarcina barkeri by Phosphate Limitation. Appl. Environ. Microbiol..

[73] Liu J.S., Schill N., van Gulik W.M., Voisard D., Marison I.W., von Stockar U. (1999). The coupling between catabolism and anabolism of Methanobacterium thermoautotrophicum in H_2_- and iron-limited continuous cultures. Enzym. Microb. Technol..

[74] Fardeau M.L., Belaich J.P. (1986). Energetics of the growth of *Methanococcus thermolithotrophicus*. Arch. Microbiol..

[75] Tsao J.H., Kaneshiro S.M., Yu S.S., Clark D.S. (1994). Continuous culture of *Methanococcus jannaschii*, an extremely thermophilic methanogen. Biotechnol. Bioeng..

[76] Mountfort D.O., Asher R.A. (1979). Effect of inorganic sulfide on the growth and metabolism of Methanosarcina barkeri strain DM. Appl. Environ. Microbiol..

[77] Pennings J.L.A., Vermeij P., Poorter L.M.I.D., Keltjens J.T., Vogels G.D. (2000). Adaptation of methane formation and enzyme contents during growth of Methanobacterium thermoautotrophicum (strain ΔH) in a fed-batch fermentor. Antonie Leeuwenhoek.

[78] Ver Eecke H.C., Akerman N.H., Huber J.A., Butterfield D.A., Holden J.F. (2013). Growth kinetics and energetics of a deep-sea hyperthermophilic methanogen under varying environmental conditions. Environ. Microbiol. Rep..

[79] Ver Eecke H.C.V., Butterfield D.A., Huber J.A., Lilley M.D., Olson E.J., Roe K.K., Evans L.J., Merkel A.Y., Cantin H.V., Holden J.F. (2012). Hydrogen-limited growth of hyperthermophilic methanogens at deep-sea hydrothermal vents. Proc. Natl. Acad. Sci. USA.

[80] Takai K., Nakamura K., Toki T., Tsunogai U., Miyazaki M., Miyazaki J., Hirayama H., Nakagawa S., Nunoura T., Horikoshi K. (2008). Cell proliferation at 122 degrees C and isotopically heavy CH_4_ production by a hyperthermophilic methanogen under high-pressure cultivation. Proc. Natl. Acad. Sci. USA.

[81] Morozova D., Wagner D. (2007). Stress response of methanogenic archaea from Siberian permafrost compared with methanogens from nonpermafrost habitats. FEMS Microbiol. Ecol..

[82] Schönheit P., Moll J., Thauer R.K. (1980). Growth parameters (K_*s*_, *μ*_*max*_, Y_*s*_) of Methanobacterium thermoautotrophicum. Arch. Microbiol..

[83] Lai M.C., Chen S.C., Shu C.M., Chiou M.S., Wang C.C., Chuang M.J., Hong T.Y., Liu C.C., Lai L.J., Hua J.J. (2002). Methanocalculus taiwanensis sp. nov., isolated from an estuarine environment. Int. J. Syst. Evol. Microbiol..

[B84-life-05-01652] 84.The latest findings show, that there is the possibility of hydrothermal vents on icy moons like Europa (e.g., [215]) or Enceladus [202], which would widen the potential temperature range for microbes in the outer Solar System.

[85] Dong X., Chen Z. (2012). Psychrotolerant methanogenic archaea: Diversity and cold adaptation mechanisms. Sci. China Life Sci..

[86] Siddiqui K.S., Cavicchioli R. (2006). Cold-Adapted Enzymes. Annu. Rev. Biochem..

[87] Feller G., Gerday C. (2003). Psychrophilic enzymes: Hot topics in cold adaptation. Nat. Rev. Microbiol..

[88] Siddiqui K., Cavicchioli R., Thomas T. (2002). Thermodynamic activation properties of elongation factor 2 (EF-2) proteins from psychrotolerant and thermophilic Archaea. Extremophiles.

[89] Thomas T., Cavicchioli R. (2000). Effect of Temperature on Stability and Activity of Elongation Factor 2 Proteins from Antarctic and Thermophilic Methanogens. J. Bacteriol..

[90] Thomas T., Kumar N., Cavicchioli R. (2001). Effects of Ribosomes and Intracellular Solutes on Activities and Stabilities of Elongation Factor 2 Proteins from Psychrotolerant and Thermophilic Methanogens. J. Bacteriol..

[91] Lim J., Thomas T., Cavicchioli R. (2000). Low temperature regulated DEAD-box RNA helicase from the antarctic archaeon, *Methanococcoides burtonii*. J. Mol. Biol..

[92] Zhou L., Liu X., Dong X. (2014). Methanospirillum psychrodurum sp. nov., isolated from wetland soil. Int. J. Syst. Evol. Microbiol..

[93] Simankova M.V., Parshina S.N., Tourova T.P., Kolganova T.V., Zehnder A.J., Nozhevnikova A.N. (2001). Methanosarcina lacustris sp. nov., a new psychrotolerant methanogenic archaeon from anoxic lake sediments. Syst. Appl. Microbiol..

[94] Zhang G., Jiang N., Liu X., Dong X. (2008). Methanogenesis from Methanol at Low Temperatures by a Novel Psychrophilic Methanogen, “Methanolobus psychrophilus” sp. nov., Prevalent in Zoige Wetland of the Tibetan Plateau. Appl. Environ. Microbiol..

[95] Chong S.C., Liu Y., Cummins M., Valentine D.L., Boone D.R. (2002). Methanogenium marinum sp. nov., a H_2_-using methanogen from Skan Bay, Alaska, and kinetics of H_2_ utilization. Antonie Van Leeuwenhoek.

[96] Zhilina T.N., Zavarzin G.A. (1987). Methanohalobium evestigatus, n. gen., n. sp. The extremely halophilic methanogenic Archaebacterium. Dokl. Akad. Nauk SSSR.

[97] Romesser J.A., Wolfe R.S., Mayer F., Spiess E., Walther-Mauruschat A. (1979). Methanogenium, a new genus of marine methanogenic bacteria, and characterization of *Methanogenium cariaci* sp. nov. and Methanogenium marisnigri sp. nov.. Arch. Microbiol..

[98] Bräuer S.L., Cadillo-Quiroz H., Ward R.J., Yavitt J.B., Zinder S.H. (2011). Methanoregula boonei gen. nov., sp. nov., an acidiphilic methanogen isolated from an acidic peat bog. Int. J. Syst. Evol. Microbiol..

[99] Maestrojuán G.M., Boone D.R., Xun L., Mah R.A., Zhang L. (1990). Transfer of Methanogenium bourgense, Methanogenium marisnigri, Methanogenium olentangyi, and Methanogenium thermophilicum to the Genus Methanoculleus gen. nov., Emendation of Methanoculleus marisnigri and Methanogenium, and Description of New Strains of Methanoculleus bourgense and Methanoculleus marisnigri. Int. J. Syst. Bacteriol..

[100] Dianou D., Miyaki T., Asakawa S., Morii H., Nagaoka K., Oyaizu H., Matsumoto S. (2001). Methanoculleus chikugoensis sp. nov., a novel methanogenic archaeon isolated from paddy field soil in Japan, and DNA-DNA hybridization among Methanoculleus species. Int. J. Syst. Evol. Microbiol..

[101] Singh N., Kendall M.M., Liu Y., Boone D.R. (2005). Isolation and characterization of methylotrophic methanogens from anoxic marine sediments in Skan Bay, Alaska: Description of *Methanococcoides alaskense* sp. nov., and emended description of Methanosarcina baltica. Int. J. Syst. Evol. Microbiol..

[102] Franzmann P.D., Springer N., Ludwig W., De Macario E.C., Rohde M. (1992). A Methanogenic Archaeon from Ace Lake, Antarctica: *Methanococcoides burtonii* sp. nov.. Syst. Appl. Microbiol..

[103] Shimada Y., Fukuda W., Akada Y., Ishida M., Nakayama J., Imanaka T., Fujiwara S. (2009). Property of cold inducible DEAD-box RNA helicase in hyperthermophilic archaea. Biochem. Biophys. Res. Commun..

[104] Saunders N.F.W., Thomas T., Curmi P.M.G., Mattick J.S., Kuczek E., Slade R., Davis J., Franzmann P.D., Boone D., Rusterholtz K. (2003). Mechanisms of Thermal Adaptation Revealed From the Genomes of the Antarctic Archaea Methanogenium frigidum and *Methanococcoides burtonii*. Genome Res..

[105] Gunnigle E., McCay P., Fuszard M., Botting C.H., Abram F., O’Flaherty V. (2013). A Functional Approach to Uncover the Low-Temperature Adaptation Strategies of the Archaeon Methanosarcina barkeri. Appl. Environ. Microbiol..

[106] Koga Y. (2012). Thermal adaptation of the archaeal and bacterial lipid membranes. Archaea (Vanc. B.C.).

[107] Nichols D.S., Miller M.R., Davies N.W., Goodchild A., Raftery M., Cavicchioli R. (2004). Cold adaptation in the Antarctic Archaeon *Methanococcoides burtonii* involves membrane lipid unsaturation. J. Bacteriol..

[108] Sprott G.D., Meloche M., Richards J.C. (1991). Proportions of diether, macrocyclic diether, and tetraether lipids in *Methanococcus jannaschii* grown at different temperatures. J. Bacteriol..

[109] Rittmann S., Herwig C. (2012). A comprehensive and quantitative review of dark fermentative biohydrogen production. Microb. Cell Fact..

[110] Nishimura N., Kitaura S., Mimura A., Takahara Y. (1992). Cultivation of thermophilic methanogen KN-15 on H_2_-CO_2_ under pressurized conditions. J. Ferment. Bioeng..

[111] Park C.B., Clark D.S. (2002). Rupture of the Cell Envelope by Decompression of the Deep-Sea Methanogen *Methanococcus jannaschii*. Appl. Environ. Microbiol..

[112] Miller J.F., Shah N.N., Nelson C.M., Ludlow J.M., Clark D.S. (1988). Pressure and Temperature Effects on Growth and Methane Production of the Extreme Thermophile *Methanococcus jannaschii*. Appl. Environ. Microbiol..

[113] Boonyaratanakornkit B., Córdova J., Park C.B., Clark D.S. (2006). Pressure affects transcription profiles of Methanocaldococcus jannaschii despite the absence of barophilic growth under gas-transfer limitation. Environ. Microbiol..

[114] Bernhardt G., Jaenicke R., Ludemann H.D. (1987). High-Pressure Equipment for Growing Methanogenic Microorganisms on Gaseous Substrates at High Temperature. Appl. Environ. Microbiol..

[115] Mayumi D., Dolfing J., Sakata S., Maeda H., Miyagawa Y., Ikarashi M., Tamaki H., Takeuchi M., Nakatsu C.H., Kamagata Y. (2013). Carbon dioxide concentration dictates alternative methanogenic pathways in oil reservoirs. Nat. Commun..

[116] Huber G., Stetter K.O. (1991). Sulfolobus metallicus, sp. nov., a Novel Strictly Chemolithoautotrophic Thermophilic Archaeal Species of Metal-Mobilizers. Syst. Appl. Microbiol..

[117] Suzuki T., Iwasaki T., Uzawa T., Hara K., Nemoto N., Kon T., Ueki T., Yamagishi A., Oshima T. (2002). Sulfolobus tokodaii sp. nov. (f. Sulfolobus sp. strain 7), a new member of the genus Sulfolobus isolated from Beppu Hot Springs, Japan. Extremophiles.

[118] Xiang X., Dong X., Huang L. (2003). Sulfolobus tengchongensis sp. nov., a novel thermoacidophilic archaeon isolated from a hot spring in Tengchong, China. Extremophiles.

[119] Schleper C., Piihler G., Kuhlmorgen B., Zillig W. (1995). Life at extremely low pH. Nature.

[120] Cadillo-Quiroz H., Yavitt J.B., Zinder S.H. (2009). Methanosphaerula palustris gen. nov., sp. nov., a hydrogenotrophic methanogen isolated from a minerotrophic fen peatland. Int. J. Syst. Evol. Microbiol..

[121] Zhilina T.N., Zavarzina D.G., Kevbrin V.V., Kolganova T.V. (2013). Methanocalculus natronophilus sp. nov., a new alkaliphilic hydrogenotrophic methanogenic archaeon from a soda lake, and proposal of the new family Methanocalculaceae. Microbiology.

[122] Rea S., Bowman J.P., Popovski S., Pimm C., Wright A.D.G. (2007). Methanobrevibacter millerae sp. nov. and Methanobrevibacter olleyae sp. nov., methanogens from the ovine and bovine rumen that can utilize formate for growth. Int. J. Syst. Evol. Microbiol..

[123] Burggraf S., Fricke H., Neuner A., Kristjansson J., Rouvier P., Mandelco L., Woese C.R., Stetter K.O. (1990). Methanococcus igneus sp. nov., a Novel Hyperthermophilic Methanogen from a Shallow Submarine Hydrothermal System. Syst. Appl. Microbiol..

[124] Jones W.J., Paynter M.J.B., Gupta R. (1983). Characterization of *Methanococcus maripaludis* sp. nov., a new methanogen isolated from salt marsh sediment. Arch. Microbiol..

[125] Robertson D.E., Noll D., Roberts M.F., Menaia J.A., Boone D.R. (1990). Detection of the osmoregulator betaine in methanogens. Appl. Environ. Microbiol..

[126] Proctor L.M., Lai R., Gunsalus R.P. (1997). The methanogenic archaeon Methanosarcina thermophila TM-1 possesses a high-affinity glycine betaine transporter involved in osmotic adaptation. Appl. Environ. Microbiol..

[127] Sowers K.R., Boone J.E., Gunsalus R.P. (1993). Disaggregation of Methanosarcina spp. and Growth as Single Cells at Elevated Osmolarity. Appl. Environ. Microbiol..

[128] Lai M.C., Sowers K.R., Robertson D.E., Roberts M.F., Gunsalus R.P. (1991). Distribution of compatible solutes in the halophilic methanogenic archaebacteria. J. Bacteriol..

[129] Roeßler M., Pflüger K., Flach H., Lienard T., Gottschalk G., Müller V. (2002). Identification of a Salt-Induced Primary Transporter for Glycine Betaine in the Methanogen Methanosarcina mazei Gö1. Appl. Environ. Microbiol..

[130] Lai M.C., Hong T.Y., Gunsalus R.P. (2000). Glycine Betaine Transport in the Obligate Halophilic Archaeon Methanohalophilus portucalensis. J. Bacteriol..

[131] Cockell C., Catling D., Wanda L., Snook K., Kepner R., Lee P., McKay C. (2000). The Ultraviolet Environment of Mars: Biological Implications Past, Present, and Future. Icarus.

[132] Beatty J., Petersen C., Chaikin A. (1999). New Solar System.

[133] Fendrihan S., Bérces A., Lammer H., Musso M., Rontó G., Polacsek T.K., Holzinger A., Kolb C., Stan-Lotter H. (2009). Investigating the Effects of Simulated Martian Ultraviolet Radiation on Halococcus dombrowskii and Other Extremely Halophilic Archaebacteria. Astrobiology.

[134] Ajon M., Fröls S., van Wolferen M., Stoecker K., Teichmann D., Driessen A.J.M., Grogan D.W., Albers S.V., Schleper C. (2011). UV-inducible DNA exchange in hyperthermophilic archaea mediated by type IV pili. Mol. Microbiol..

[135] Fröls S., Gordon P.M.K., Panlilio M.A., Duggin I.G., Bell S.D., Sensen C.W., Schleper C. (2007). Response of the Hyperthermophilic Archaeon Sulfolobus solfataricus to UV Damage. J. Bacteriol..

[136] Fröls S., Ajon M., Wagner M., Teichmann D., Zolghadr B., Folea M., Boekema E.J., Driessen A.J.M., Schleper C., Albers S.V. (2008). UV-inducible cellular aggregation of the hyperthermophilic archaeon Sulfolobus solfataricus is mediated by pili formation. Mol. Microbiol..

[137] Fröls S., White M.F., Schleper C. (2009). Reactions to UV damage in the model archaeon Sulfolobus solfataricus. Biochem. Soc. Trans..

[138] Kral T., Shina N. Sensitivity of methanogens to ultraviolet radiation under aerobic and anaerobic conditions. Proceedings of the 2012 Astrobiology Science Conference.

[139] Sinha N., Kral T.A. (2013). Methanogen Sensitivity to Ultraviolet Radiation: Implications for Life on Mars. Meteorit. Planet. Sci. Suppl..

[140] Stan-Lotter H., Legat A., Fendrihan S., Leuko S., Gruber C., Radax C., Pfaffenhuemer M., Weidler G., Rittmann S. (2004). Haloarchaeal survival over geological times and the detection of extraterrestrial halite—Implications for the search for life on Mars. Proceedings of the Third European Workshop on Exo-Astrobiology.

[141] Radax C., Pfaffenhuemer M., Wieland H., Rittmann S., Leuko S., Weidler G., Gruber C., Stan-Lotter H. (2002). Microbes in rock salt: How to find out what is in there. Proceedings of the First European Workshop on Exo-Astrobiology.

[142] Rittmann S., Legat A., Fendrihan S., Stan-Lotter H. (2004). Viability and morphology of Halobacterium species following desiccation—Implications for contaminants on Mars. Proceedings of the Third European Workshop on Exo-Astrobiology.

[143] Leuko S., Weidler G., Rittmann S., Stan-Lotter H. (2004). LIVE/DEAD Kit: A powerful tool to detect haloarchaeal survival (and life?) in unknown environmental samples. Proceedings of the Third European Workshop on Exo-Astrobiology.

[144] Liu C.T., Miyaki T., Aono T., Oyaizu H. (2008). Evaluation of methanogenic strains and their ability to endure aeration and water stress. Curr. Microbiol..

[145] Schirmack J., Alawi M., Wagner D. (2015). Influence of Martian regolith analogs on the activity and growth of methanogenic archaea, with special regard to long-term desiccation. Extrem. Microbiol..

[146] Solar Dynamics Observatory/Atmospheric Imaging Assembly (2010). The Sun at 304 Angstroms. http://sdo.gsfc.nasa.gov/assets/img/browse/2010/08/19/20100819_003221_4096_0304.jpg.

[147] International Astronomical Union (2006). Eight Planets and New Solar System Designations. http://apod.nasa.gov/apod/ap060828.html.

[148] Tate K. (2015). Dwarf Planets in the Solar System. http://i.space.com/images/i/000/023/868/original/dwarf-planets-121120b-02.jpg?1353517196.

[149] National Aeronautics and Space Administration (2004). Moons of the Solar System. http://solarsystem.nasa.gov/multimedia/gallery/Many_Moons-browse.jpg.

[150] Archinal B.A., A’Hearn M.F., Bowell E., Conrad A., Consolmagno G.J., Courtin R., Fukushima T., Hestroffer D., Hilton J.L., Krasinsky G.A. (2011). Report of the IAU Working Group on Cartographic Coordinates and Rotational Elements: 2009. Celest. Mech. Dyn. Astron..

[151] Chamberlin A. Planetary Satellite Physical Parameters. http://ssd.jpl.nasa.gov/?sat_phys_par.

[152] National Aeronautics and Space Administration (2010). Ten Cool Things Seen in the First Year of LRO. http://www.nasa.gov/mission_pages/LRO/news/first-year.html.

[153] Schenk P. (2010). Atlas of the Galilean Satellites.

[154] Chamberlin A. Planets and Pluto: Physical Characteristics. http://ssd.jpl.nasa.gov/?planet_phys_par.

[155] Spohn T., Breuer D., Johnson T. (2014). Encyclopedia of the Solar System.

[156] Thomas P.C., Parker J.W., McFadden L.A., Russell C.T., Stern S.A., Sykes M.V., Young E.F. (2005). Differentiation of the asteroid Ceres as revealed by its shape. Nature.

[157] Delitsky M.L., Lane A.L. (1998). Ice chemistry on the Galilean satellites. J. Geophys. Res..

[158] Moore J.M., Chapman C.R., Bierhaus E.B., Greeley R., Chuang F.C., Klemaszewski J., Clark R.N., Dalton J.B., Hibbitts C.A., Schenk P.M., Bagenal F., Dowling T.E., McKinnon W.B. (2004). Callisto. Jupiter. The Planet, Satellites and Magnetosphere.

[159] Jaumann R., Kirk R.L., Lorenz R.D., Lopes R.M.C., Stofan E., Turtle E.P., Keller H.U., Wood C.A., Sotin C., Soderblom L.A., Brown R.H., Lebreton J.P., Waite J.H. (2010). Geology and Surface Processes on Titan. Titan from Cassini-Huygens.

[160] Thomas P.C. (2010). Sizes, shapes, and derived properties of the saturnian satellites after the Cassini nominal mission. Icarus.

[161] Spencer J.R., Pearl J.C., Segura M., Flasar F.M., Mamoutkine A., Romani P., Buratti B.J., Hendrix A.R., Spilker L.J., Lopes R.M.C. (2006). Cassini Encounters Enceladus: Background and the Discovery of a South Polar Hot Spot. Science.

[162] Porco C.C., Helfenstein P., Thomas P.C., Ingersoll A.P., Wisdom J., West R., Neukum G., Denk T., Wagner R., Roatsch T. (2006). Cassini Observes the Active South Pole of Enceladus. Science.

[163] Iess L., Stevenson D.J., Parisi M., Hemingway D., Jacobson R.A., Lunine J.I., Nimmo F., Armstrong J.W., Asmar S.W., Ducci M. (2014). The Gravity Field and Interior Structure of Enceladus. Science.

[164] Buie M.W., Grundy W.M., Young E.F., Young L.A., Stern S.A. (2006). Orbits and Photometry of Pluto’s Satellites: Charon, S/2005 P1, and S/2005 P2. AJ.

[165] Stern S.A., Trafton L.M., Barucci M.A., Boehnhardt H., Cruikshank D.P., Morbidelli A., Dotson R. (2008). On the Atmospheres of Objects in the Kuiper Belt. The Solar System Beyond Neptune.

[166] Vance S., Bouffard M., Choukroun M., Sotin C. (2014). Ganymede’s internal structure including thermodynamics of magnesium sulfate oceans in contact with ice. Planet. Space Sci..

[167] Lollar B.S., McCollom T.M. (2006). Geochemistry: Biosignatures and abiotic constraints on early life. Nature.

[168] Guzmán-Marmolejo A., Segura A., Escobar-Briones E. (2013). Abiotic Production of Methane in Terrestrial Planets. Astrobiology.

[169] White W. (2015). Isotope Geochemistry.

[170] Mahaffy P.R., Webster C.R., Atreya S.K., Franz H., Wong M., Conrad P.G., Harpold D., Jones J.J., Leshin L.A., Manning H. (2013). Abundance and Isotopic Composition of Gases in the Martian Atmosphere from the Curiosity Rover. Science.

[171] Hoekzema N., Gwinner K., Grieger B., Markiewicz W.J., Keller H., Hoffmann H., Meima J.A., Neukum G. (2006). The Dust Scale Height of the Martian Atmosphere around Pavonis Mons from Hrsc Stereo Images. AAS/Division for Planetary Sciences Meeting Abstracts.

[172] Williams D.R. Mars Fact Sheet. http://nssdc.gsfc.nasa.gov/planetary/factsheet/marsfact.html.

[173] Williams D.R. Earth Fact Sheet. http://nssdc.gsfc.nasa.gov/planetary/factsheet/earthfact.html.

[174] Lowell P. (1911). Mars and Its Canals.

[175] Villanueva G.L., Mumma M.J., Novak R.E., Käufl H.U., Hartogh P., Encrenaz T., Tokunaga A., Khayat A., Smith M.D. (2015). Strong water isotopic anomalies in the martian atmosphere: Probing current and ancient reservoirs. Science.

[176] Ojha L., Wilhelm M.B., Murchie S.L., McEwen A.S., Wray J.J., Hanley J., Massé M., Chojnacki M. (2015). Spectral evidence for hydrated salts in recurring slope lineae on Mars. Nature Geoscience.

[177] Feldman W.C., Pathare A., Maurice S., Prettyman T.H., Lawrence D.J., Milliken R.E., Travis B.J. (2011). Mars Odyssey neutron data: 2. Search for buried excess water ice deposits at nonpolar latitudes on Mars. J. Geophys. Res. (Planets).

[178] Hand E. (2012). Curiosity set to weigh in on Mars methane puzzle. Nature.

[B179-life-05-01652] 179.The potential CH_4_ signal was in fact coming from CO_2_ ice.

[180] Webster C.R., Mahaffy P.R., Atreya S.K., Flesch G.J., Farley K.A., Team M.S. (2013). Low Upper Limit to Methane Abundance on Mars. Science.

[181] Zahnle K., Freedman R.S., Catling D.C. (2011). Is there methane on Mars?. Icarus.

[182] Fonti S., Marzo G.A. (2010). Mapping the methane on Mars. A & A.

[B183-life-05-01652] 183.Minor sources are, e.g., UV radiation induced generation of CH_4_ from organic chemicals and released CH_4_ from CH_4_ hydrates (*i.e.*, clathrates), that may be a record of past biological activity.

[184] Catling D.C., Cockell C.S., McKay C.P. Ultraviolet Radiation on the Surface of Mars. Proceedings of the Fifth International Conference on Mars.

[185] Gaidos E.J., Nealson K.H., Kirschvink J.L. (1999). Life in Ice-Covered Oceans. Science.

[186] McKinnon W.B., Pappalardo R.T., Khurana K.K., Pappalardo R.T., McKinnon W.B., Khurana K.K. (2009). Europa: Perspectives on an Ocean World. Europa.

[187] Oren A. (2001). The bioenergetic basis for the decrease in metabolic diversity at increasing salt concentrations: Implications for the functioning of salt lake ecosystems. Hydrobiologia.

[188] Hussmann H., Sohl F., Spohn T. (2006). Subsurface oceans and deep interiors of medium-sized outer planet satellites and large trans-neptunian objects. Icarus.

[189] Turse C., Leitner J.J., Firneis M.G., Schulze-Makuch D. (2013). Simulations of Prebiotic Chemistry under Post-Impact Conditions on Titan. Life.

[190] Hand K.P., Chyba C.F., Priscu J.C., Carlson R.W., Nealson K.H., Pappalardo R.T., McKinnon W.B., Khurana K.K. (2009). Astrobiology and the Potential for Life on Europa. Europa.

[191] Chamberlin A. Planetary Satellite Mean Orbital Parameters. http://ssd.jpl.nasa.gov/?sat_elem.

[192] Spencer J. (2009). Planetary science: Enceladus with a grain of salt. Nature.

[193] McKinnon W.B. (2015). Effect of Enceladus’s rapid synchronous spin on interpretation of Cassini gravity. Geophys. Res. Lett..

[194] Postberg F., Kempf S., Schmidt J., Brilliantov N., Beinsen A., Abel B., Buck U., Srama R. (2009). Sodium salts in E-ring ice grains from an ocean below the surface of Enceladus. Nature.

[195] Taubner R.S., Leitner J., Firneis M., Hitzenberger R. (2015). Modelling the Interior Structure of Enceladus Based on the 2014’s Cassini Gravity Data. J. Orig. Life Evol. Biosph..

[196] Schubert G., Anderson J.D., Travis B.J., Palguta J. (2007). Enceladus: Present internal structure and differentiation by early and long-term radiogenic heating. Icarus.

[197] Postberg F. Sodium salts in cryo-volcanic ice particles—Evidence for liquid water on Enceladus. Proceedings of the CHARM Meeting.

[198] Postberg F., Schmidt J., Hillier J., Kempf S., Srama R. (2011). A salt-water reservoir as the source of a compositionally stratified plume on Enceladus. Nature.

[199] Thomas P., Tajeddine R., Tiscareno M., Burns J., Joseph J., Loredo T., Helfenstein P., Porco C. (2016). Enceladus’s measured physical libration requires a global subsurface ocean. Icarus.

[200] Bouquet A., Mousis O., Waite J.H., Picaud S. (2015). Possible evidence for a methane source in Enceladus’ ocean. Geophys. Res. Lett..

[201] Waite J.H., Lewis W.S., Magee B.A., Lunine J.I., McKinnon W.B., Glein C.R., Mousis O., Young D.T., Brockwell T., Westlake J. (2009). Liquid water on Enceladus from observations of ammonia and ^40^Ar in the plume. Nature.

[202] Hsu H.W., Postberg F., Sekine Y., Shibuya T., Kempf S., Horányi M., Juhász A., Altobelli N., Suzuki K., Masaki Y. (2015). Ongoing hydrothermal activities within Enceladus. Nature.

[203] Howett C., Spencer J., Verbiscer A. (2014). Enceladus’ enigmatic heat flow. AAS/Division for Planetary Sciences Meeting Abstracts.

[204] Howett C.J.A., Spencer J.R., Pearl J., Segura M. (2011). High heat flow from Enceladus’ south polar region measured using 10–600 cm^-1^ Cassini/CIRS data. J. Geophys. Res. (Planets).

[205] Hansen C.J., Shemansky D.E., Esposito L.W., Stewart A.I.F., Lewis B.R., Colwell J.E., Hendrix A.R., West R.A., Waite J.H., Teolis B. (2011). The composition and structure of the Enceladus plume. Geophys. Res. Lett..

[206] Horányi M., Juhász A., Morfill G.E. (2008). Large-scale structure of Saturn’s E-ring. Geophys. Res. Lett..

[207] Kempf S., Srama R., Moragas-Klostermeyer G., Postberg F., Horányi M., Schmidt J., Spahn F. The Structure of Saturn’s E ring as seen by Cassini CDA. Proceedings of the 2011 EPSC-DPS Joint Meeting.

[208] Schubert G., Sohl F., Hussmann H., Pappalardo R.T., McKinnon W.B., Khurana K.K. (2009). Interior of Europa. Europa.

[209] Taubner R.S., Leitner J., Firneis M., Hitzenberger R. Estimations on the Interior of Small Icy Bodies in the Solar System (EGU2014-7338). Presented at the European Geosciences Union General Assembly.

[210] Kivelson M.G., Khurana K.K., Russell C.T., Volwerk M., Walker R.J., Zimmer C. (2000). Galileo Magnetometer Measurements: A Stronger Case for a Subsurface Ocean at Europa. Science.

[211] Carr M.H., Belton M.J.S., Chapman C.R., Davies M.E., Geissler P., Greenberg R., McEwen A.S., Tufts B.R., Greeley R., Sullivan R. (1998). Evidence for a subsurface ocean on Europa. Nature.

[212] Greenberg R., Geissler P., Hoppa G., Tufts B.R. (2002). Tidal-Tectonic Processes and Their Implications for the Character of Europa’s Icy Crust. Rev. Geophys..

[213] Brown M.E., Hand K.P. (2013). Salts and Radiation Products on the Surface of Europa. AJ.

[214] Roth L., Saur J., Retherford K.D., Strobel D.F., Feldman P.D., McGrath M.A., Nimmo F. (2014). Transient Water Vapor at Europa’s South Pole. Science.

[215] Zolotov M.Y., Kargel J.S., Pappalardo R.T., McKinnon W.B., Khurana K.K. (2009). On the Chemical Composition of Europa’s Icy Shell, Ocean, and Underlying Rocks. Europa.

[216] McCollom T.M. (1999). Methanogenesis as a potential source of chemical energy for primary biomass production by autotrophic organisms in hydrothermal systems on Europa. J. Geophys. Res. Planets.

[217] Niemann H.B., Atreya S.K., Demick J.E., Gautier D., Haberman J.A., Harpold D.N., Kasprzak W.T., Lunine J.I., Owen T.C., Raulin F. (2010). Composition of Titan’s lower atmosphere and simple surface volatiles as measured by the Cassini-Huygens probe gas chromatograph mass spectrometer experiment. J. Geophys. Res. (Planets).

[218] Raulin F. (2008). Astrobiology and habitability of Titan. Space Sci. Rev..

[219] Raulin F., McKay C., Lunine J., Owen T., Brown R.H., Lebreton J.P., Waite J.H. (2010). Titan’s Astrobiology. Titan from Cassini-Huygens.

[220] Fulchignoni M., Ferri F., Angrilli F., Ball A.J., Bar-Nun A., Barucci M.A., Bettanini C., Bianchini G., Borucki W., Colombatti G. (2005). *In situ* measurements of the physical characteristics of Titan’s environment. Nature.

[221] Clarke D.W., Ferris J.P., Whittet D.C.B. (1997). Chemical Evolution on Titan: Comparisons to the Prebiotic Earth. Planetary and Interstellar Processes Relevant to the Origins of Life.

[222] Balucani N., Cartechini L., Bergeat A., Casavecchia P., Volpi G.G., Ehrenfreund P., Angerer O., Battrick B. (2001). Laboratory studies on the formation of CN containing molecules in the atmosphere of Titan and prebiotic Earth. Proceedings of the First European Workshop on Exo-/Astro-Biology.

[223] Neish C.D., Somogyi Á., Imanaka H., Lunine J.I., Smith M.A. (2008). Rate Measurements of the Hydrolysis of Complex Organic Macromolecules in Cold Aqueous Solutions: Implications for Prebiotic Chemistry on the Early Earth and Titan. Astrobiology.

[224] He C., Smith M.A. (2014). Identification of nitrogenous organic species in Titan aerosols analogs: Implication for prebiotic chemistry on Titan and early Earth. Icarus.

[225] Sotin C., Jaumann R., Buratti B.J., Brown R.H., Clark R.N., Soderblom L.A., Baines K.H., Bellucci G., Bibring J.P., Capaccioni F. (2005). Release of volatiles from a possible cryovolcano from near-infrared imaging of Titan. Nature.

[226] O’Brien D.P., Lorenz R.D., Lunine J.I. (2005). Numerical calculations of the longevity of impact oases on Titan. Icarus.

[227] Iess L., Jacobson R.A., Ducci M., Stevenson D.J., Lunine J.I., Armstrong J.W., Asmar S.W., Racioppa P., Rappaport N.J., Tortora P. (2012). The Tides of Titan. Science.

[228] Kerr R.A. (2012). Cassini Spies an Ocean Inside Saturn’s Icy, Gassy Moon Titan. Science.

[229] Sotin C., Mitri G., Rappaport N., Schubert G., Stevenson D., Brown R.H., Lebreton J.P., Waite J.H. (2010). Titan’s Interior Structure. Titan from Cassini-Huygens.

[230] Buratti B., Faulk S., Mosher J., Baines K., Brown R., Clark R., Nicholson P. (2011). Search for and limits on plume activity on Mimas, Tethys, and Dione with the Cassini Visual Infrared Mapping Spectrometer (VIMS). Icarus.

[B231-life-05-01652] 231.There is an atmosphere indicating some material outflowing from the surface both at Dione and Rhea (J.H. Waite, personal communication, July, 2015).

[232] Tajeddine R., Rambaux N., Lainey V., Charnoz S., Richard A., Rivoldini A., Noyelles B. (2014). Constraints on Mimas’ interior from Cassini ISS libration measurements. Science.

[233] Gaeman J., Hier-Majumder S., Roberts J.H. (2012). Sustainability of a subsurface ocean within Triton’s interior. Icarus.

[234] Soderblom L.A., Becker T.L., Kieffer S.W., Brown R.H., Hansen C.J., Johnson T.V., Kirk R.L., Shoemaker E.M., Cook A.F. (1990). Triton’s geyser-like plumes—Discovery and basic characterization. Science.

[235] International Astronomical Union (2006). RESOLUTION B5—Definition of a Planet in the Solar System.

[236] Küppers M., O’Rourke L., Bockelée-Morvan D., Zakharov V., Lee S., von Allmen P., Carry B., Teyssier D., Marston A., Müller T. (2014). Localized sources of water vapour on the dwarf planet (1)Ceres. Nature.

[237] Witze A. (2015). Mystery haze appears above Ceres’s bright spots. Nat. NEWS.

[238] Robuchon G., Nimmo F. (2011). Thermal evolution of Pluto and implications for surface tectonics and a subsurface ocean. Icarus.

[239] Rhoden A.R., Henning W., Hurford T.A., Hamilton D.P. (2015). The interior and orbital evolution of Charon as preserved in its geologic record. Icarus.

[B240-life-05-01652] 240.Large TNOs show a higher amount of rocky material, especially Quaoar with a mean density of 4200±1300 kg m^-3^ [270].

[241] Allen C.C., Morris R.V., Lindstrom D.J., Lindstrom M.M., Lockwood J.P. JSC Mars-1—Martian regolith simulant. Lunar and Planetary Institute Technical Report, Proceedings of the Lunar and Planetary Science Conference.

[B242-life-05-01652] 242.According to the reaction *Fe*^0^ + 2*H*^+^ → *Fe*^2+^ + *H*_2_, zero-valent iron could serve as reactant to produce H_2_ [67].

[243] Djordjevic S., Mickol R.L., Kral T.A. Simulating Martian Conditions: Methanogen Survivability during Freeze-Thaw Cycles. Proceedings of the Lunar and Planetary Science Conference.

[244] Mickol R.L., Kral T.A., Laird S.K. Mesophile Methanogen Survival Under Freeze/Thaw Cycles. Lunar and Planetary Institute Technical Report, Proceedings of the Lunar and Planetary Science Conference.

[B245-life-05-01652] 245.*Methanosarcina spec. SMA-16, SMA-23, M. soligelidi, Methanobacterium spec. MC-20, M. barkeri, and M. frigidum*.

[246] McKay C.P., Porco C.C., Altheide T., Davis W.L., Kral T.A. (2008). The Possible Origin and Persistence of Life on Enceladus and Detection of Biomarkers in the Plume. Astrobiology.

[B247-life-05-01652] 247.Found in the Columbia River basalts and basalts in the Twin Falls area of Idaho, respectively [246].

[248] Stevens T.O., McKinley J.P. (1995). Lithoautotrophic Microbial Ecosystems in Deep Basalt Aquifers. Science.

[249] Chapelle F.H., O’Neill K., Bradley P.M., Methé B.A., Ciufo S.A., Knobel L.L., Lovley D.R. (2002). A hydrogen-based subsurface microbial community dominated by methanogens. Nature.

[B250-life-05-01652] 250.Found in the 3-to 4-km-deep fracture in the 2.7-billion-year-old Ventersdorp Supergroup metabasalt [251].

[251] Lin L.H., Wang P.L., Rumble D., Lippmann-Pipke J., Boice E., Pratt L.M., Sherwood Lollar B., Brodie E.L., Hazen T.C., Andersen G.L. (2006). Long-Term Sustainability of a High-Energy, Low-Diversity Crustal Biome. Science.

[252] McKay C.P., Khare B.N., Amin R., Klasson M., Kral T.A. (2012). Possible sources for methane and C_2_-C_5_ organics in the plume of Enceladus. Planet. Space Sci..

[253] Parashar S., Kral T.A. (2010). Possibility of Methanogens on Enceladus. LPI Contrib..

[254] Taubner R.S., Rittmann S., Leitner J., Schleper C., Firneis M., Hitzenberger R. Assessing the feasibility to cultivate methanogens under Enceladus-like conditions reservoir. Presented at the 14th European Workshop on Astrobiology. Presented at the 14th European Workshop on Astrobiology.

[255] Preston L.J., Dartnell L.R. (2014). Planetary habitability: Lessons learned from terrestrial analogues. Int. J. Astrobiol..

[256] Morris R.V., Blake D.F., Bish D., Ming D.W., Agresti D.G., Treiman A.H., Steele A., Amundsen H.E.F., Amase Team A Terrestrial Analogue from Spitsbergen (Svalbard, Norway) for the Comanche Carbonate at Gusev Crater, Mars. Lunar and Planetary Institute Technical Report, Proceedings of the Lunar and Planetary Science Conference.

[257] Gómez-Gómez F., Rodriguez-Manfredi J.A., Perez L., Prieto-Ballesteros O., Amils R., Gomez-Elvira J. Martian Habitability Studies in Two Field Earth Analogues: The Permafrost in the Imuruk Lake Basaltic Field (Alaska) and the Atacama Desert. Proceedings of the 38th COSPAR Scientific Assembly.

[258] Shtarkman Y.M., Koçer Z.A., Edgar R., Veerapaneni R.S., D’Elia T., Morris P.F., Rogers S.O. (2013). Subglacial Lake Vostok (Antarctica) Accretion Ice Contains a Diverse Set of Sequences from Aquatic, Marine and Sediment-Inhabiting Bacteria and Eukarya. PLoS ONE.

[259] Patil U., Muskan K. (2009). Essentials of Biotechnology.

[260] Fuchs G., Eitinger T., Schlegel H. (2007). Allgemeine Mikrobiologie.

[261] Krajete A., Herwig C., Rittmann S., Seifert A., Bernacchi S. (2014). Method and System for Producing Methane Using Methanogenic Microorganisms and Applying Specific Nitrogen Concentrations in the Liquid Phase.

[262] Plaut J.J., Barabash S., Bruzzone L., Dougherty M., Erd C., Fletcher L., Gladstone R., Grasset O., Gurvits L., Hartogh P. Jupiter Icy Moons Explorer (JUICE): Science Objectives, Mission and Instruments. Proceedings of the Lunar and Planetary Science Conference.

[263] Grasset O., Dougherty M.K., Coustenis A., Bunce E.J., Erd C., Titov D., Blanc M., Coates A., Drossart P., Fletcher L.N. (2013). JUpiter ICy moons Explorer (JUICE): An ESA mission to orbit Ganymede and to characterise the Jupiter system. Planet. Space Sci..

[264] European Space Agency/Science and Robotic Exploration (2014). JUICE Definition Study Report (Red Book).

[265] National Aeronautics and Space Administration (2015). VALKYRIE: Phase 2. https://astrobiology.nasa.gov/astep/projects/nra/nnh11zda001n-astep/valkyrie-phase-2/.

[266] Karl D.M., Beversdorf L., Björkman K., Church M.J., Martinez A., Delong E.F. (2008). Aerobic production of methane in the sea. Nat. Geosci..

[267] Metcalf W.W., Griffin B.M., Cicchillo R.M., Gao J., Janga S.C., Cooke H.A., Circello B.T., Evans B.S., Martens-Habbena W., Stahl D.A. (2012). Synthesis of Methylphosphonic Acid by Marine Microbes: A Source for Methane in the Aerobic Ocean. Science.

[268] Carini P., White A., Campbell E., Giovannoni S. (2014). Methane production by phosphate-starved SAR11 chemoheterotrophic marine bacteria. Nat. Commun..

[269] Lecar M., Podolak M., Sasselov D., Chiang E. (2006). On the Location of the Snow Line in a Protoplanetary Disk. ApJ.

[270] Fraser W.C., Brown M.E. (2010). Quaoar: A Rock in the Kuiper Belt. ApJ.

